# Supplementary material for: Inflammatory Micro-environment Contributes to Stemness Properties and Metastatic Potential of HCC via the NF-κB/miR-497/SALL4 Axis
Source: Mol Ther Oncolytics. 2019 Sep 10;15:79–90. doi: 10.1016/j.omto.2019.08.009 (PMC6804787; doi:10.1016/j.omto.2019.08.009)
Supplement: Document S1. Figures S1–S6 and Tables S1–S3 [file mmc1.pdf]

OMTO, Volume *15*

Supplemental Information

Inflammatory Micro-environment Contributes to Stemness Properties and Metastatic Potential of HCC via the NF-κB/miR-497/SALL4 Axis

Bixing Zhao, Yingchao Wang, Xionghong Tan, Kun Ke, Xiaoyuan Zheng, Fei Wang, Shubing Lan, Naishun Liao, Zhixiong Cai, Yingjun Shi, Youshi Zheng, Yongping Lai, Lili Wang, Qin Li, Jingfeng Liu, Aimin Huang, and Xiaolong Liu

Supplementary information

Supplementary Figures

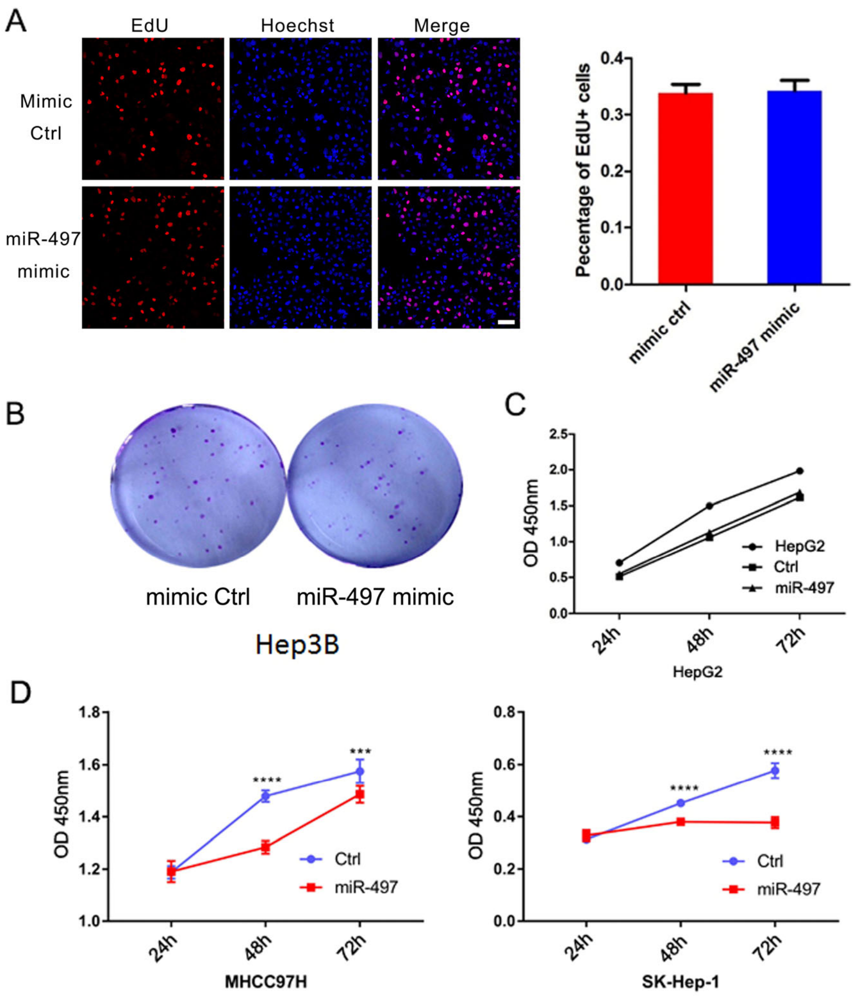

Figure S1. Effect of miR-497 on proliferation of hepatocellular carcinoma cells. (A) Hep3B cells were transfected with mimic Control or miR-497 mimic. The EdU proliferation assay was performed 48 h after transfection. Representative image and ratio of EdU-positive Hep3B cells were shown. Scale bar, 100μm. (B) The colony formation assay was conducted to determine the colony-forming growth of cells and the colonies were captured on the 14th day after seeding. (C-D) Proliferation was analyzed by CCK-8 assay in miR-497 over-expressing HepG2 (C) MHCC97H (D) and SK-Hep-1 (D) cells. ***P<0.001, ****P<0.0001.

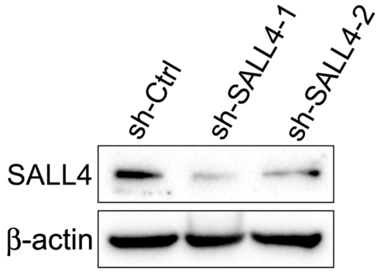

Figure S2. SALL4 knockdown verification at the protein level by western blot analysis. Hep3B cells were infected with lentiviruses to introduce constitutively active shRNA control or shRNA against SALL4, and western blot was carried out 4 days after infection.

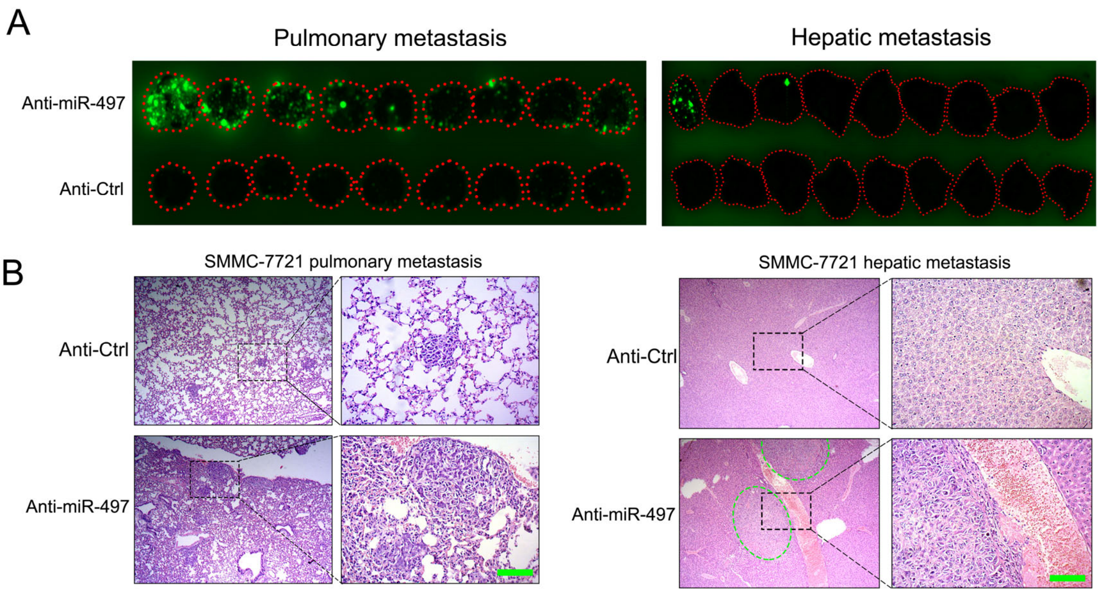

Figure S3. miR-497 suppressed SMMC-7721 cell lung and hepatic metastasis in mouse model (A) Lung and hepatic metastasis of anti-control and anti-miR497 SMMC-7721 cells in NOD/SCID mice at 6 weeks after tail vein implantation. Lungs and livers from the NOD/SCID mice were dissected and the presence of HCC metastasis and detected by green fluorescence signal. (B) Representative H&E-stained sections of the lung and liver tissues collected from anti-Ctrl and Anti-miR-497 groups. Scale bars, 100μm.

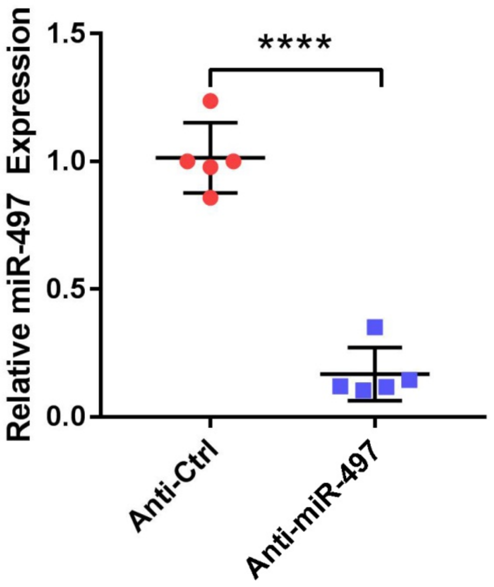

Figure S4. miR-497 expression verification in lung metastasis nodules. Lung metastasis nodules from the NOD/SCID mice (Anti-Ctrl and Anti-miR-497 group) were dissected and the levels of miR-497 were analyzed using real-time qRT-PCR. ****$P<0.0001$.

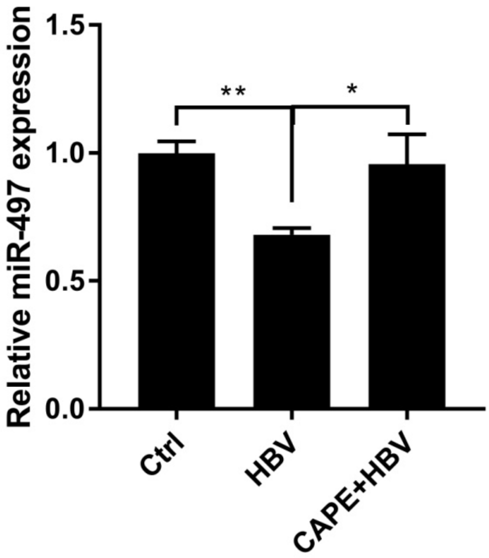

Figure S5. HBV suppresses miR-497 expression in a NF-κB dependent manner. HepG2 cells were transfected with pAAV-HBV2.1, then treated with 25μg/ml of CAPE, for 24 h, and miR-497 was detected by real-time qRT-PCR. *P<0.05, **P<0.01.

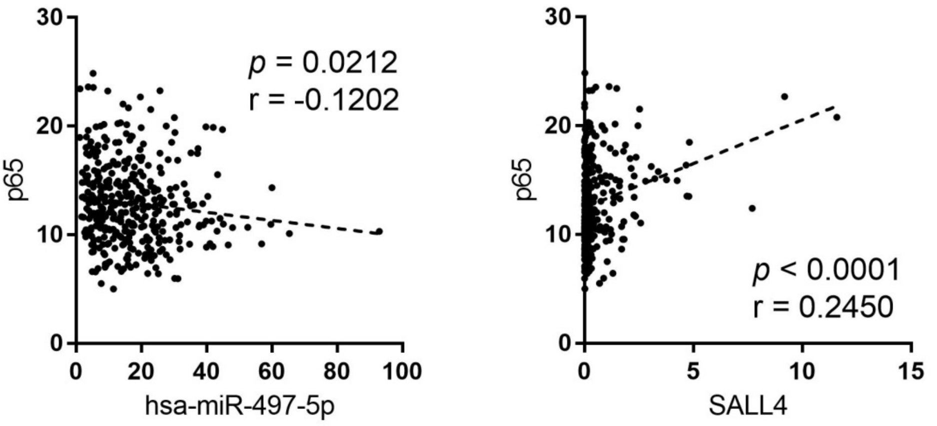

Figure S6. TCGA data analysis of the correlation of p65 with miR-497 and p65 with SALL4. Inverse correlation of miR-497 expression with p65 expression. Positive correlation of SALL4 expression with p65 expression.

Supplementary Table 1. Sequences of the insertion fragments of Luciferase reporter plasmid.

Name	WT/Mutant	Insertion sequence
SALL4 3'-UTR luciferase reporter	WT	GGGAGAACTTGCGTGGAAGGAGCAATGCAGACAC<br>AGTGAAATCTCTAGAATCTGCTTTGTTTTGTAAGAA<br>CTCATCTCCTCCTGTTTTCTTTTTCTTACTGATATGC<br>AAATGATGTTTACTACGTTGGTTGTGACCACAACCT<br>CAGGCAAGTGCTACAATCACGATTGTTGCTATGCT<br>GCTTTGCAAAAAGTTGAAAAAATAAAAAAAAAATGC<br>ATACCA AAAA
	Mutant	GGGAGAACTTGCGTGGAAGGAGCAATGCAGACAC<br>AGTGAAATCTCTAGAATCTGCTTTGTTTTGTAAGAA<br>CTCATCTCCTCCTGTTTTCTTTTTCTTACTGATATGC<br>AAATGATGTTTACTACGTTGGTTGTGACCACAACCT<br>CAGGCAAGTGCTACAATCACGATTGTTGCTAACGA<br>CGATTGCAAAAAGTTGAAAAAATAAAAAAAAAATGC<br>ATACCAAAAA
PGL3-miR497-promoter	WT	CCCCACCCTAGGGATCCCCTGAGCTGAGTTCC<br>TACAGAGGGAAGATGGTCCAATCTTACTACACT<br>GTGAGCTCATCCCCATGGTCCGTCGCCTTCCA<br>GTTGCCTGCTCAGCCCGTCCCTGGTTCCTCCC<br>AAACGTTTTTGGGGGCCATGTTTGCCTTTTAAG<br>GCTTCTCTATCCCCCCGCTCCTGGAGGTGGTG<br>CTGGGGTCTTCCCAGCACTGC
	D1	CCCCACCCTATGAGCTGAGTTCCTACAGAGGG<br>AAGATGGTCCAATCTTACTACACTGTGAGCTCA<br>TCCCCATGGTCCGTCGCCTTCCAGTTGCCTGC<br>TCAGCCCGTCCCTGGTTCCTCCCAAACGTTTT<br>TGGGGGCCATGTTTGCCTTTTAAGGCTTCTCTA<br>TCCCCCCGCTCCTGGAGGTGGTGCTGGGGTC<br>TTCCCAGCACTGC
	D2	CCCCACCCTAGGGATCCCCTGAGCTGAGTTCC<br>TACAGAGGGAAGATGGTCCAATCTTACTACACT<br>GTGAGCTCATCCCCATGGTCCGTCGCCTTCCA<br>GTTGCCTGCTCAGCCCGTCCCTGGTTCCTCCC<br>AAACGTTTTTGGGGGCCATGTTTGCCTTTTAAG<br>GCTTCTCTATCCCCCCGCTCCTGGAGGTGGTG<br>CTCCAGCACTGC

Supplementary Table 2. Primers for qRT-PCR

Name	F/R	Primer Sequence
SALL4	F	CGCCCCGTGTGTCATGTAGTGAAC
	R	TCCGAGAACAGCCGCACTGAGATGGAAG
CDH1	F	GCCGAGAGCTACACGTTCA
	R	GACCGGTGCAATCTTCAAA
Vimentin	F	GACGCCATCAACACCGAGTT
	R	CTTTGTCGTTGGTTAGCTGGT
Nanog	F	CTATAACTGTGGAGAGGAAT
	R	AGTGGTCTGCTGTATTAC
Oct4	F	GTATTCAGCCAAACGACCAT
	R	CTTCCTCCACCCACTTCT
Bmi-1	F	GCTTTGTGGAGGGTACTTCATT
	R	AGGACAATACTTGCTGGTCTC
GAPDH	F	AGCCACATCGCTCAGACAC
	R	GCCCAATACGACCAAATCC

Supplementary Table 3: Univariate and Multivariate Analysis of Factors Associated with Overall survival.

**Clinical Variables**	**Case Number**	**Univariate analysis**		**Multivariate analysis**	
		**HR (95% CI)**	***P* value**	**HR (95% CI)**	***P* value**
miR-497 (Low vs High)	38/37	0.388 (0.160-0.945)	**0.037**	0.286 (0.112-0.730)	**0.009**
Gender (M vs F)	68/7	2.621 (0.353-19.452)	0.346		
Age-yr (≥ 55 vs < 55)	36/39	1.460 (0.640-3.331)	0.369		
Tumor size (> 5 vs ≤ 5 cm)	26/49	5.519 (2.328-13.087)	**0.001**	5.040 (2.075-12.240)	**0.001**
Vascular invasion (Yes vs No)	44/31	0.522 (0.214-1.269)	0.151		
AFP (> 400 vs ≤ 400 ng/ml)	21/54	3.145 (1.384-7.147)	**0.006**	2.618 (1.091-6.284)	**0.031**
HBV (Positive vs Negative)	68/7	0.405 (0.055-3.003)	0.376		
Tumor number (Solitary vs Multiple)	60/15	1.372 (0.541-3.480)	0.506		
Cirrhosis (Yes vs No)	69/6	2.514 (0.745-8.486)	0.138		
TNM stage (I/II vs III/IV)	55/20	3.578 (1.574-8.130)	**0.002**	1.743 (0.682-4.451)	0.246
