# Supplementary material for: Inflammatory Micro-environment Contributes to Stemness Properties and Metastatic Potential of HCC via the NF-κB/miR-497/SALL4 Axis
Source: Mol Ther Oncolytics. 2019 Sep 10;15:79–90. doi: 10.1016/j.omto.2019.08.009 (PMC6804787; doi:10.1016/j.omto.2019.08.009)
Supplement: Document S2. Article plus Supplemental Information [file mmc2.pdf]

Molecular Therapy
Oncolytics
Original Article

Inflammatory Micro-environment Contributes to Stemness Properties and Metastatic Potential of HCC via the NF-κB/miR-497/SALL4 Axis

Bixing Zhao,[1,3,4] Yingchao Wang,[1,3,4] Xionghong Tan,[1,4] Kun Ke,[1,4] Xiaoyuan Zheng,[1,4] Fei Wang,[1,4] Shubing Lan,[1,4] Naishun Liao,[1,3,4] Zhixiong Cai,[1,3,4] Yingjun Shi,[1,4] Youshi Zheng,[1,3,4] Yongping Lai,[5] Lili Wang,[6] Qin Li,[1,3,4] Jingfeng Liu,[1,3,4] Aimin Huang,[2,4] and Xiaolong Liu[1,3,4]

[1]The United Innovation of Mengchao Hepatobiliary Technology Key Laboratory of Fujian Province, Mengchao Hepatobiliary Hospital of Fujian Medical University, Fuzhou 350025, P.R. China; [2]Department of Pathology, School of Basic Medical Sciences, Fujian Medical University, Fuzhou 350108, P.R. China; [3]Mengchao Med-X Center, Fuzhou University, Fuzhou 350116, P.R. China; [4]The Liver Center of Fujian Province, Fujian Medical University, Fuzhou 350025, P.R. China; [5]Department of Hepatobiliary and Pancreatic Surgery, Mengchao Hepatobiliary Hospital of Fujian Medical University, Fuzhou 350025, P.R. China; [6]Department of Diagnostic Radiology, Fujian Medical University Union Hospital, Fuzhou 350001, P.R. China

Increasing evidence has demonstrated the essential role of inflammatory micro-environment in tumorigenesis and tumor progression. Some cancer cells in tumor maintain typical stemness properties and, with the capacity of self-renewal, are thought to be crucial for the initiation and maintenance of tumors as well as their metastasis. Although both inflammatory micro-environment and stemness properties played crucial roles in tumor initiation and development, currently it is still unclear whether and how the inflammatory micro-environment promotes cancer stemness properties. Here, we show the first evidence that the inflammatory micro-environment promotes the stemness properties and metastatic potential of hepatocellular carcinoma (HCC) via the NF-κB/miR-497/SALL4 axis. We discover that miR-497 directly targets SALL4, negatively regulates its expression, and further inhibits the self-renewal and metastasis of HCC; more importantly, inflammatory factor TNF-α inhibits the expression of miR-497 via NF-kB-mediated negative transcriptional regulation and simultaneously upregulates the expression of SALL4 and promotes the self-renewal and metastasis phenotypes of HCC cells. Moreover, lower expression of miR-497 is significantly associated with poor prognosis in HCC patients. Taken together, our findings not only revealed a novel signaling pathway (NF-κB/miR-497/SALL4 axis) to connect inflammation with stemness properties, and clarified the molecular mechanisms underlying the inflammation-mediated self-renewal and metastasis phenotypes, but also provided novel molecular targets for developing new anticancer strategies.

INTRODUCTION

Hepatocellular carcinoma (HCC) is one of the most common malignant tumors worldwide, especially in East Asia, including China, Korea, and Japan.[1] Due to the highly aggressive nature of HCCs, which are extremely resistant to traditional treatments such as chemotherapy and radiation, the 5-year survival rate of HCC is extremely poor.

Currently, a remarkable number of proposed poor prognostic signatures for HCC are related to "stemness," and cells with the expression of hepatic stem/progenitor-cell-related markers in HCCs—cancer stem cells, with the capacity to self-renew and undergo aberrant differentiation[2]—have been tightly associated with the aggressive clinical behaviors.[3]

The inflammatory responses play critical roles at all stages of tumor development, including initiation, promotion, malignant conversion, invasion, and metastasis.[4] However, the relationship between chronic hepatic inflammation and cancer stem cell generation remains obscure. The driving molecular events in inflammation-triggered tumor self-renewal remain to be elucidated.

SALL4 (sal-like 4) is a transcription factor that plays essential roles in maintaining the self-renewal and pluripotency of embryonic stem cells (ESCs).[5,6] Recent studies in HCC show that high expression of SALL4 is associated with aggressive behavior and poor prognosis of HCC in clinical investigations.[7–9] As a stem cell biomarker in liver cancers, SALL4 regulates cell proliferation, apoptosis, migration/invasion, drug resistance, and stemness by targeting a variety of genes. Although the function of SALL4 in regulating the stemness of HCC

Received 5 May 2019; accepted 15 August 2019;
https://doi.org/10.1016/j.omto.2019.08.009.

Correspondence: Aimin Huang, Department of Pathology, School of Basic Medical Sciences, Fujian Medical University, 1 Xueyuan Road, Fuzhou 350108, P.R. China.
E-mail: aimin@fjmu.edu.cn
Correspondence: Xiaolong Liu, The United Innovation of Mengchao Hepatobiliary Technology Key Laboratory of Fujian Province, Mengchao Hepatobiliary Hospital of Fujian Medical University, 312 Xihong Road, Fuzhou 350025, P.R. China.
E-mail: xiaoloong.liu@gmail.com

This is an open access article under the CC BY license (http://creativecommons.org/licenses/by/4.0/).

has been very clear, there is no clear evidence that SALL4 is a key signal network node to connect the inflammation with stemness properties. Interestingly, multiple studies show that SALL4-positive HCC patients were also associated with a significantly high frequency of hepatitis B virus (HBV) infection,[8–10] suggesting that SALL4 is closely related to chronic inflammation.

MicroRNAs (miRNAs) are a class of endogenous and small noncoding regulatory RNAs, which mainly recognize complementary sequences in the 3′ UTRs of their target genes and lead to mRNA degradation or translation inhibition.[11] Aberrant expression of certain miRNAs has been found to be closely correlated with a variety of biological processes, including maintaining cancer stem cells,[12] suggesting that certain miRNAs might function as oncogenes or tumor suppressors.[13–15]

miR-497 is dysregulated in various cancers via a variety of mechanisms, including transcriptional alterations, epigenetic alterations, and genomic alterations. It acts as a key modulator of proliferation, differentiation, apoptosis, angiogenesis, migration, and invasion. So far, miR-497 mainly works as a tumor suppressor gene. However, whether miR-497 is involved in regulating the stemness of cancer cells has not been reported. Using bioinformatics analysis and luciferase assay, we discover that miR-497 directly targets the 3′ UTR of SALL4.

Although extensive studies have been conducted, the exact molecular mechanisms that link the inflammation micro-environment and stemness properties in HCC still remain unclear. In this study, we aimed to investigate the regulatory mechanism of miR-497 in the progression of HCC and explore the role of the miR-497/SALL4 axis in the process of inflammation-triggered self-renewal and metastasis phenotypes. The study presented here would reveal a new pathway that participated in the signal transduction of connecting inflammation with stemness properties and metastatic potential and, in the meantime, would provide a novel explanation for the roles that inflammation micro-environment played in HCC self-renewal and progression. Targeting this signaling pathway might help the development of novel strategies for HCC treatment.

RESULTS

miR-497 Directly Targets SALL4 in HCC Cells

SALL4 is overexpressed in cancer cells and affects multiple cellular processes that are involved in tumorigenesis, tumor growth, and tumor progression. To further expand SALL4-related signaling networks and explore the molecular mechanisms of SALL4 in regulating the progression of HCC, potential regulatory miRNAs of SALL4 were predicted using TargetScan and miRanda. miR-497 was chosen as a potential regulator of SALL4, which may bind to the SALL4 3′ UTR in positions 175–181, and the binding sequence is relatively conserved among different species (Figure 1A).

To determine whether SALL4 is directly regulated by miR-497, the sequence of the SALL4 3′ UTR was cloned into the pMIR-REPORT vector, and the potential binding site was mutated to construct the reporter gene vector as control (Figure 1B). A luciferase reporter assay revealed that the co-transfection of a miR-497 mimic significantly inhibited the activity of firefly luciferase reporter with the wild-type 3′ UTR of SALL4, whereas this effect was abrogated when the predicted 3′ UTR binding site was mutated (Figure 1B). At the mean time, when endogenous miR-497 was stably knocked down by the lentiviral miR-497 inhibitor (anti-miR497), the activity of the luciferase reporter gene was significantly enhanced; however, the reporter gene of the mutant vector did not change. These results further demonstrated that miR-497 specifically and directly binds to the 3′ UTR of SALL4.

To demonstrate the silencing effects of miR-497 on SALL4, we performed transient transfection studies by using a chemically synthesized miR-497 mimic. Compared with the control group, transfection of Hep3B cells with the miR-497 mimic caused a remarkable upregulation of the miR-497 level and resulted in the inhibition of SALL4 in both mRNA (Figure 1C) and protein (Figure 1D) levels. To investigate the consequences of miR-497 inhibition, we transduced HepG2 cells with either a control or a miRZip antisense miR-497-encoded lentivirus. miR-497 inhibition by anti-miR-497 in HepG2 cells dramatically increased the expression of SALL4 (Figures 1C and 1D). Moreover, we found that SALL4 levels were reversely correlated to miR-497 levels in 50 human HCC tissues (Figure 1E). Additionally, the inverse correlation between miR-497 and SALL4 expression was further confirmed in HCC cell lines (Figure 1F). Together, these data indicate that miR-497 may negatively regulate SALL4 expression by directly targeting its 3′ UTR.

miR-497 Regulates HCC Metastasis and Self-Renewal

It has been shown that SALL4 is a marker for the progenitor subclass of HCC cells with aggressive phenotypes.[8,16] We wondered whether miR-497 may alter the malignant phenotypes of HCC cells, including migration/invasion ability, proliferation, and self-renewal. To determine the impact of miR-497 on HCC cell proliferation, Hep3B cells were transfected with miR-497 mimic, and an EdU incorporation and cell proliferation assay and a colony formation assay were performed; the results show that miR-497 has no effects on proliferation of Hep3B cells (Figures S1A and S1B). Next, cell proliferation was monitored by the Cell Counting Kit-8 (CCK-8) in other HCC cell lines. Interestingly, miR-497 inhibits proliferation of SK-Hep-1 and MHCC97H cells (Figure S1D), while it seems to have no effects on the proliferation of HepG2 cells (Figure S1C). Thus, miR-497 regulates HCC cell proliferation in a cell-line-dependent manner.

To elucidate the role of miR-497 in HCC metastasis, the effects of miR-497 on the migration and invasion of HCC cells were analyzed initially *in vitro*. Transwell assays showed that both the migratory and invasive activities of HCC cells were suppressed by miR-497 expression but were promoted when cellular miR-497 was neutralized by anti-miR-497 (Figure 2A). The wound healing assay was used to further confirm this response, and the data also proved that miR-497 negatively regulated HCC cells' migration in both miR-497-overexpressed Hep3B cells and miR-497-knockdown HepG2 cells (Figure 2B). As epithelial-mesenchymal transition (EMT) has a central role in cancer metastasis, we further examined the expression levels of

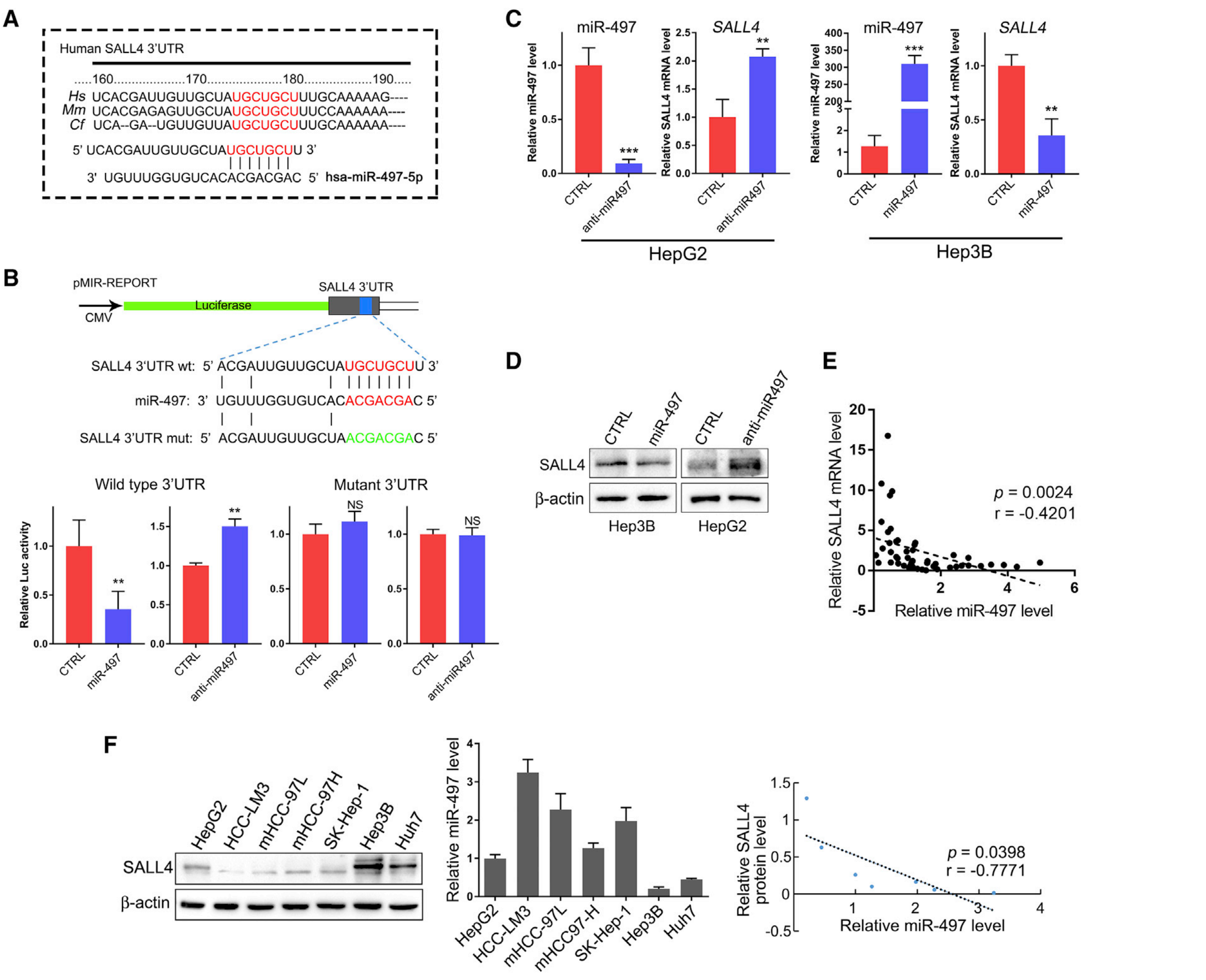

Figure 1. miR-497 Directly Targets SALL4

(A) Putative binding sites of miR-497 in the SALL4 3′ UTR of different species. (B) SALL4 3′ UTR firefly luciferase activity in HEK293T cells. Upper panel: the sequences of wild-type and mutant 3′ UTR show the segments cloned into the CMV-promoter-driven luciferase reporter plasmid. Lower panel: pMIR-REPORT and β-gal gene expression vector, together with miR-497 mimic, anti-miR497, or control vector as indicated, were transfected into 293T cells. Reporter gene activity was determined and normalized in relation to the co-transfected β-gal activity. The bars represent the average ± mean from three independent experiments. (C) miR-497 and SALL4 mRNA expression in miR-497-overexpressing Hep3B cells and miR-497-knockdown HepG2 cells was analyzed by real-time RT-PCR assay. (D) Protein expression of SALL4 by western blotting analysis in transfected Hep3B and HepG2 cells. (E) Expression levels of miR-497 and SALL4 were negatively correlated in 50 HCC samples, as measured by real-time PCR. The relative expression of miR-497 and SALL4 values (normalized to 18S rRNA) was subjected to Pearson correlation analysis. (F) Detection of the expression of miR-497 and SALL4 in different HCC cell lines by real-time RT-PCR and western blot, respectively; scatterplot indicates the negative correlation between miR-497 and SALL4 expression. Pearson's coefficient test was performed to assess statistical significance. *$p < 0.05$; **$p < 0.01$; ***$p < 0.001$.

EMT-related factors by qPCR and western blot. The data show that the knockdown of miR-497 down-regulated the expression of E-cadherin/*CDH1* and upregulated the expression of vimentin in both mRNA and protein levels (Figures 2D and 2E). In contrary, the knockdown of miR-497 had an opposite effect on the expression of E-cadherin and vimentin protein in HepG2 cells (Figure 2E). In summary, these data suggest that miR-497 regulated the process of EMT, resulting in the suppression of the migratory ability of HCC *in vitro*.

SALL4 is suggested as a stem cell marker in liver cancer that regulates the self-renewal of liver cancer cells;[7] therefore, we further investigated the effect of miR-497 on self-renewal of HCC cells via tumor spheroid formation assay. Phenotypically, miR-497 inhibition in HepG2 cells resulted in the significant activation of spheroid formation capacities (Figure 2C) with upregulation of NANOG, OCT4, and Bmi-1 expression in both mRNA and protein levels (Figures 2D and 2E). Conversely, when Hep3B was transiently transfected with miR-497 mimic, the spheroid formation capacities were apparently suppressed (Figure 2C) and the expression of stem cell markers NANOG, OCT4, and Bmi-1 were increased as expected (Figure 2E). To sum up, the aforementioned data suggest a pivotal role for miR-497 in regulating the self-renewal and metastasis of HCC cells.

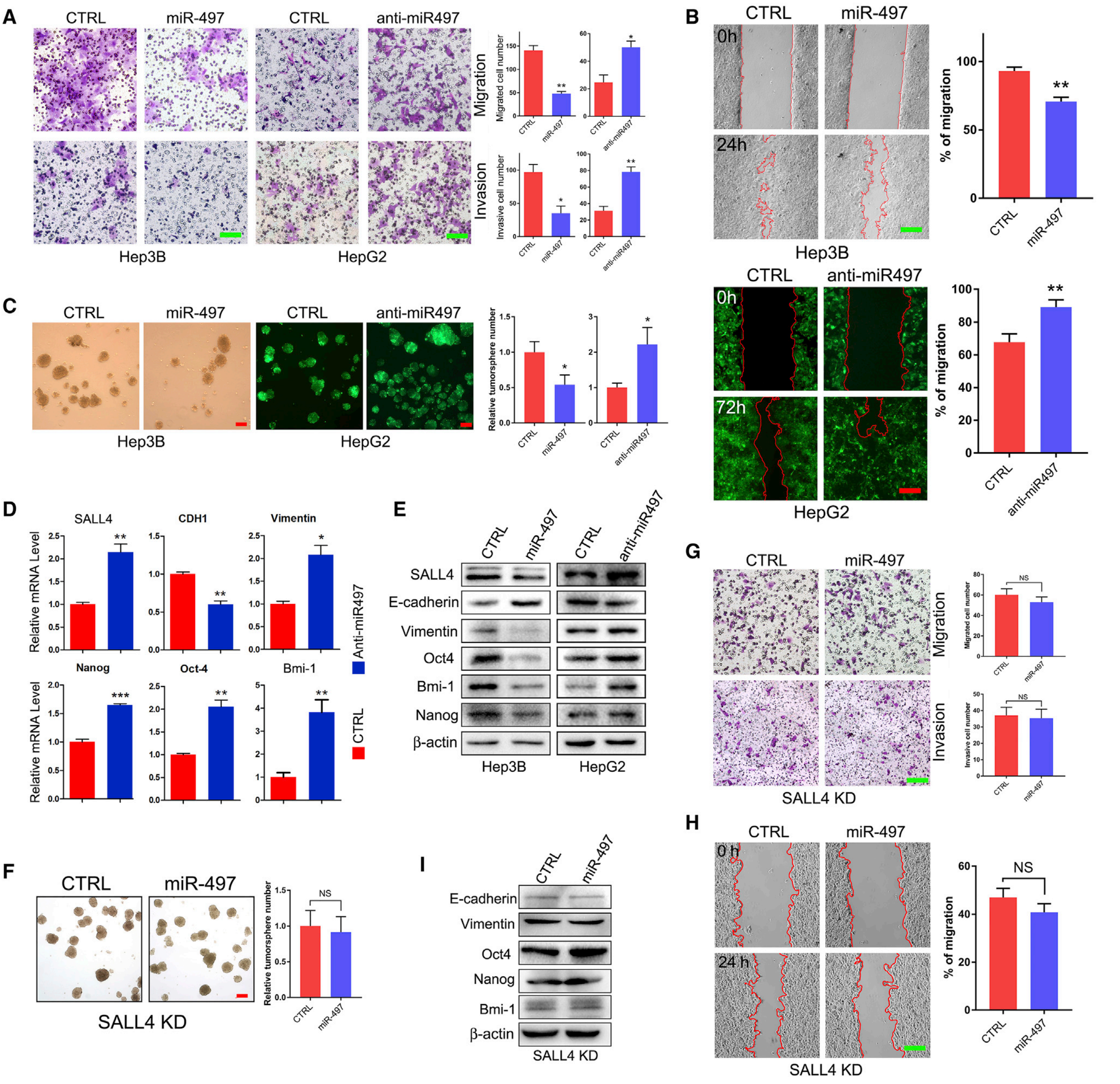

Figure 2. miR-497 Regulates HCC Metastasis and Self-Renewal

(A) Hep3B and HepG2 cells transduced with miR-497 mimic, anti-miR497 vector, or control vector as indicated were subjected to cell migration and invasion assays. Migrated/invaded cells in fields were quantified, and representative photographs are shown. Data are represented as mean ± SEM. *p < 0.05; **p < 0.01. (B) Wound healing assay was performed to determine the cell migratory capacity. (C) Tumor sphere-forming capacity in HCC cell lines as indicated was analyzed by tumor sphere formation assay; the right panel indicates statistical results as means ± SEM. (D) qRT-PCR analysis of the mRNA expression of SALL4, CDH1, vimentin, Nanog, Oct4, and Bmi-1 in HepG2/Ctrl and HepG2/Anti-miR-497 cells. (E) Western blot analysis of SALL4, E-cadherin, vimentin, Oct4, Bmi-1, and Nanog expression in Hep3B/Ctrl and Hep3B/miR-497 cells, as well as in HepG2/Ctrl and HepG2/anti-miR-497 cells. (F) SALL4 knockdown abolished sphere formation ability reduced by miR-497. Representative sphere formation is indicated in the left panel. Relative tumor sphere numbers were calculated as means ± SEM (right panel). (G and H) SALL4 knockdown abolished migration and invasion capacity down-regulated by miR-497 as analyzed by Transwell assay (G) and wound healing assay (H). (I) The expression of E-cadherin, vimentin, Oct4, Bmi-1, and Nanog was detected in SALL4 KD/Ctrl and SALL4 KD/miR497 HCC cells. *p < 0.05; **p < 0.01. Scale bars, 100 μm.

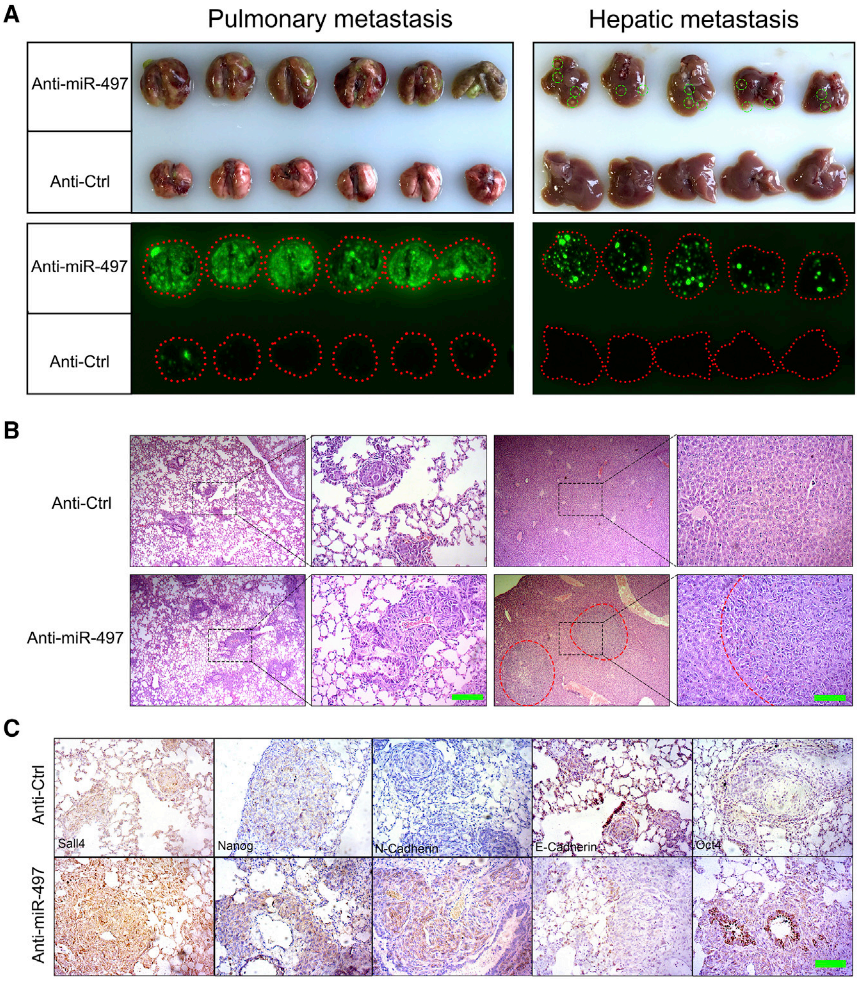

Figure 3. miR-497 Suppressed HCC Metastasis *In Vivo*

(A) Lung and hepatic metastasis of control and anti-miR497 SK-Hep-1 cells in NOD/SCID mice at 6 weeks after tail vein implantation. Lungs and livers from the NOD/SCID mice were dissected, and the presence of HCC metastasis was detected by green fluorescence signal. (B) Representative H&E-stained sections of the lung and liver tissues collected from control and anti-miR-497 groups. Scale bars, 100 μm. (C) Representative IHC staining pictures of the lung sections of control and anti-miR-497 groups. Scale bars, 50 μm.

To further confirm that miR-497 regulates HCC metastasis and self-renewal via targeting SALL4, we next investigated whether SALL4 knockdown can abolish the effect of miR-497 in HCC cells. Then, SALL4 was stably knocked down in Hep3B cells by using a lentiviral-short hairpin RNA (shRNA) construct (Figure S2). In SALL4-knockdown cells, further miR-497 overexpression did not affect the self-renewal feature of this cell (Figure 2F). Transwell and wound healing assays also indicated that miR-497 was unable to affect the invasion and migration abilities of HCC in SALL4-knockdown cells (Figures 2G and 2H); meanwhile, the expression of EMT and stemness factors had no significant change (Figure 2I). These results suggest that miR-497 may repress HCC metastasis and self-renewal by targeting SALL4.

miR-497 Regulates HCC Metastasis *In Vivo*

To further validate the aforementioned findings *in vivo*, a tail vein injection model was conducted with metastatic HCC cell lines SMMC-7721 and SK-Hep-1. Before vein injection, both HCC cell lines were infected with anti-miR497 or control lentivirus (GFP^+). Then, 5×10^6 cells were injected through the caudal vein. 5 weeks after injection, the mice were killed by cervical decapitation. Lung and liver were dissected, and metastatic nodules were examined using the Bio-Rad Imaging System by detecting the GFP fluorescence of metastatic HCC cells. As shown in Figure 3A, miR-497 knockdown dramatically increased the metastatic nodules in both the lung and liver of the SK-Hep-1 metastatic tumor model. In the Anti-miR-497 group, intense GFP fluorescence was observed in all lung and liver. While in the control group, both fluorescence intensity and metastatic nodule number were significantly lower than those in the anti-miR-497 group. Histologic analysis further confirms that miR-497 knockdown (Figure S4) remarkably facilitates the metastasis of HCC in xenograft transplantation mouse models of metastasis. The same results were also observed in the SMMC-7721 model (Figures S3A and S3B) and reconfirmed the anti-metastasis role of miR-497 *in vivo*.

Afterward, we further investigated whether miR-497 expression was associated with SALL4 and its downstream targets in lung metastasis lesions. As expected, the level of SALL4 expression was strongly increased in miR-497 knockdown metastatic lesions when compared with control lung metastatic lesions (Figure 3B). EMT and stem cell markers E-cadherin, vimentin, OCT-4, and Nanog were detected by immunohistochemistry (IHC) at the same time, and the results were kept as the same as *in vitro* (Figures 3C and 2E). Therefore, these data from mouse models complement and reconfirm our findings from cell-line models, which strongly support the role of miR-497 as a tumor-suppressing gene through direct targeting of SALL4.

Down-Regulation of miR-497 in HCC Patients Is Associated with Worse Prognosis

Previous studies have shown that miR-497 was down-regulated in HCC tissue samples.[17,18] To further investigate the biological significance of miR-497 in HCC, we analyzed the correlation between miR-497 levels and the clinical characteristics of HCC patients in this study. Seventy-five samples of HCC tissues were subjected to qRT-PCR for miR-497 expression analysis. The median value of all 75 cases was chosen as the cutoff point for separating the miR-497 low-expression and miR-497 high-expression groups. As shown in

Table 1. Correlation between miR-497-5p Expression and Clinical-Pathological Features in HCC

Variable	Category	miR-497 High	miR-497 Low	p Value
Gender	male	33	35	0.970
	female	4	3	
Age	≥55	17	19	0.725
	<55	20	19	
Tumor size	>5 cm	12	14	0.688
	≤5 cm	25	24	
Vascular invasion	yes	18	26	0.082
	no	19	12	
AFP (ng/mL)	>400	9	12	0.484
	≤400	28	26	
HBV	positive	31	37	0.043*
	negative	6	1	
Tumor number	solitary	34	26	0.011*
	multiple	3	12	
Cirrhosis	yes	33	36	0.376
	no	4	2	
TNM stage	I	17	7	0.011*
	II–IV	20	31	
Recurrence	yes	14	25	0.015*
	no	23	13	

AFP, alpha fetoprotein.
*$p < 0.05$.

Table 1, the low expression of miR-497 was prominently associated with positive HBV infection (p = 0.043), multiple tumor numbers (p = 0.011), advanced tumor node metastasis (TNM) stage (p = 0.011), and recurrence (p = 0.015). The Kaplan-Meier plots revealed an association of lower miR-497 levels with shorter overall survival (OS; p = 0.035; Figure 4A), recurrence-free survival (RFS; p = 0.0308; Figure 4A), and higher cumulative incidence of recurrence (p = 0.0463; Figure 4B). Multivariate Cox regression analysis further confirmed miR-497 as an independent risk factor for OS (hazard ratio [HR], 0.286; p = 0.009; Table S3). Moreover, to analyze the difference expression of miR-497 in patients with different clinical features, the chi-square test was conducted, and the results revealed that lower miR-497 levels were significantly associated with the presence of metastasis (Figure 4C), TNM stage (Figure 4D), and tumor numbers (Figure 4E). Thus, our results indicate that low expression of miR-497 is correlated with malignant clinicopathologic characteristics in HCC.

NF-κB Directly Trans-suppresses miR-497 under Chronic Inflammatory Conditions

HCC was recognized as an inflammation-driven disease, and 90% of HCCs were developed on the chronic liver inflammation background.[19] The clinical data show that the expression of miR-497 is also associated with HBV infection (Table 1). Thus, we wondered whether chronic inflammation may regulate the expression of miR-497. Therefore, the chronic inflammation was induced in HepG2 cells by HBV infection or pro-inflammatory cytokine (interleukin [IL]-6, IL-17, or tumor necrosis factor alpha [TNF-α]) treatment. As shown in Figure 5A, transfection with the pAAV/HBV1.2 plasmid in HepG2 cells significantly down-regulated the expression of miR-497. Pro-inflammatory cytokine treatment (TNF-α, IL-6, and IL-17) also significantly reduced the miR-497 expression level (Figure 5A). The nuclear factor κB (NF-κB) signaling pathway was considered one of the key pathways to modulate hepatic inflammation,[20] and NF-κB could be activated by various stimuli and cellular stresses, including TNF-α. Therefore, we next explored whether chronic inflammation regulates the expression of miR-497 through the NF-κB signaling pathway. As shown in Figure 5B and Figure S5, when HCC cells were treated with CAPE, a specific inhibitor of NF-κB, TNF-α and HBV lost the ability to regulate the expression of miR-497. As a transcription factor complex, inflammatory signaling induces the formation of NF-κB heterodimers (p65/p50) to locate into the nucleus and then to bind with binding sites of specific gene promoters, subsequently to trans-activate or trans-suppress the expression of target gene. These binding sites share highly conserved nucleotides with the consensus sequence (GGGRNNYYC). Therefore, the sequence in the promoter region of miR-497 was analyzed, and two potential p65 binding sequences (GGGRNNYYC) were identified (GGGATCCCC in −227 through −219 and GGGGTCTTC in −41 through −43) (Figure 5C). An electrophoretic mobility shift assay (EMSA) was carried out, and the data show that a strong gel-shift signal was detected when the first p65-binding site probe was added in the SMMC-7721 nuclear extracts. However, there was almost no gel-shift signal for the probe of the second p65-binding site (Figure 5E).

To further confirm that NF-κB binds to the promoter region of miR-497 and then transcriptionally inhibits the expression of miR-497, the promoter region of miR-497 was cloned into the luciferase reporter vector and deletion mutations that delete the two potential binding sites were also constructed, respectively. As expected, luciferase reporter gene assays showed that the relative luciferase activity of miR-497 promoter was significantly elevated when the first p65-binding site (−227 bp) was deleted, while no significant change was observed when the second binding site (−41 bp) was deleted (Figure 5D). Taken together, these data demonstrate that p65 binds to the −227 to −219 region of the miR-497 promoter and inhibits the transcription of miR-497.

Inflammation Micro-environment Induces HCC Stemness through the NF-κB/miR-497/SALL4 Axis

Previous studies have shown that the expression of SALL4 in HCC is closely associated with HBV infection.[8–10] Our aforementioned data also showed that inflammatory signal trans-suppresses miR-497 and that miR-497 targets SALL4 and suppresses metastasis and self-renewal of HCC. Therefore, we hypothesized whether the inflammatory micro-environment regulates HCC self-renewal through the miR-497-SALL4 pathway. To confirm this hypothesis, the expression

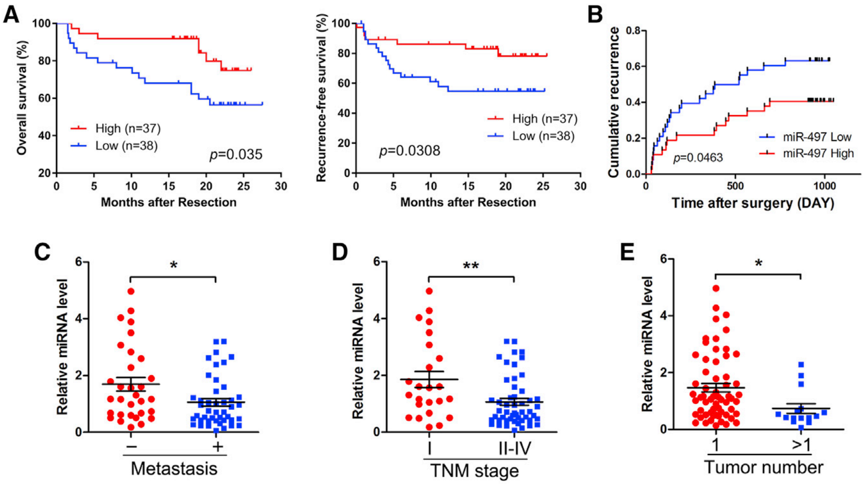

Figure 4. Down-Regulation of miR-497 Is Associated with Worse Prognosis

(A) The Kaplan-Meier plot represents OS and RFS of HCC patients based on their expression of miR-497. The levels of miR-497 were analyzed using real-time qRT-PCR, and the median value of all 75 cases was chosen as the cutoff point for separating the miR-497 low-expression and miR-497 high-expression groups. (B) Cumulative incidence of tumor recurrence curves for different miR-497 expression groups. (C) Metastatic HCC displayed lower miR-497 expression levels. The absence (n = 31) and presence (n = 44) of venous invasion are indicated with a minus sign (−) and plus sign (+), respectively. (D) Expression of miR-497 in HCC with TNM stages II–IV (n = 51) were significantly lower compared with the TNM I group (n = 24). (E) Expression of miR-497 in HCC with multiple tumors (n = 15) was significantly lower compared with the solitary tumor (n = 60) group. The central horizontal line represents the mean value; the error bars represent the SEM. *p < 0.05; **p < 0.01.

of SALL4 was detected upon inflammation induction in HCC cells; combined with the results in Figure 5A, it could clearly show that inflammation induced by HBV or TNF-α significantly down-regulated miR-497 expression (Figure 5A) and upregulated the expression of SALL4, at both the mRNA and protein levels (Figures 6A and 6C). Similarly, the NF-κB inhibitor CAPE abrogates the upregulation of SALL4 induced by TNF-α (Figure 6B). Furthermore, inhibition of the NF-κB pathway dramatically abrogated the TNF-α-induced expression of self-renewal regulatory factors such as NANOG, OCT4, and SOX2 (Figure 6C). Thus, TNF-α down-regulated miR-497 and upregulated the expression of SALL4 and other self-renewal regulatory factors via the NF-κB signaling pathway.

The TNF-α-induced self-renewal phenotype was further investigated by tumor sphere formation assay to make evident the ability of TNF-α in promoting the self-renewal of HCC cells (Figure 6F). To further confirm that TNF-α regulated HCC self-renewal in a miR-497-dependent manner, the miR-497 stable-knockdown HCC cells were treated with TNF-α, and the tumor sphere formation assay was carried out. The results showed that TNF-α could significantly enhance the tumor sphere formation ability of control cell lines but not in the anti-miR-497 group, indicating that TNF-α improves HCC self-renewal specifically depending on miR-497. Western blot analysis further confirmed that TNF-α treatment failed to increase the expression of SALL4 and self-renewal regulatory factors (NANOG, SOX, and OCT4) in anti-497 HCC cells (Figure 6D).

To further verify that inflammation regulates the malignant phenotype of HCC via miR-497 *in vivo*, the HCC cell metastatic tumor model was established, combined with TNF-α injection. As shown in Figure 6E, TNF-α injection significantly increased the tumor formation rate of lung metastases (5/6 versus 2/6) and the number of metastatic lesions (p = 0.048), whereas in miR-497-knockdown mice, the lung metastasis rates of untreated and TNF-α-treated groups were 5/6 and 6/6, and there was no significant difference between the number of metastatic lesions, which was consistent with the aforementioned *in vitro* data. These data demonstrate that TNF-α promotes the malignant phenotype of liver cancer through miR-497. Additionally, a statistically significant inverse correlation between p65 and miR-497 and a positive correlation between p65 and SALL4 were observed in HCC tissues from the The Cancer Genome Atlas (TCGA) dataset (Figure S6).

Overall, these results demonstrate that TNF-α-activated NF-κB binds directly to the promoter region of miR-497 and down-regulates miR-497, which, in turn, upregulates the expression of SALL4 and other self-renewal regulatory factors and finally contributes to the self-renewal and metastasis malignant features of HCC (Figure 6G).

DISCUSSION

Numerous studies show that miRNA is important in the regulation of gene expression networks, and miRNAs are continuously being shown to act as both oncogenes and tumor suppressors. Aberrant expression of miRNAs in HCC has been profiled in many studies.[21–23] A few studies have shown that miR-497 may suppresses angiogenesis, metastasis, and proliferation in HCC.[17,18] In addition, miR-497 was found to target specific oncogenes such as VEGFA, IGF-1R, CHEK1, and Raf-1.[17,18,24–26] Those studies all show that miR-497 may act as a tumor suppressor, and it has been well known that miRNAs would exhibit cell-type or tissue-specific actions in a context-dependent manner, which may explain the differences between those studies. Similarly, here, we show that miR-497 serves as a tumor suppressor gene by targeting SALL4 and regulates the metastasis and stemness of HCC. The proinflammatory cytokine TNF-α activates the NF-κB signaling pathway, leading to nuclear import of p65 and binding to the p65-binding site on the miR-497 promoter, which, in turn, inhibits the transcription of miR-497, thereby upregulating the expression of SALL4 and eventually promoting EMT and the self-renewal phenotype of HCC (Figure 6G).

Multiple miRNAs targeting SALL4 have been found in recent years. In glioma, miR-107 suppressed cell growth and induced apoptosis

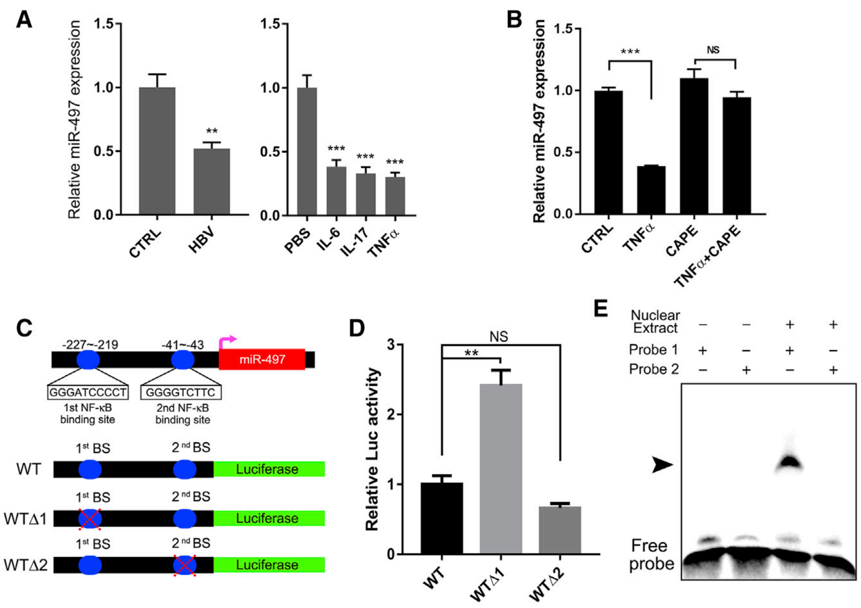

Figure 5. NF-κB Directly Trans-suppresses miR-497 under Chronic Inflammatory Conditions

(A) Inflammation suppresses the expression of miR-497. HepG2 cells were transfected with pAAV-HBV2.1 or treated with 100 ng/mL IL-6, IL-17, or TNF-α as indicated for 24 h, and the levels of miR-497 were analyzed using real-time qRT-PCR. (B) TNF-α down-regulated miR-497 in an NF-κB-dependent manner. HepG2 cells were pre-treated with 25 μg/mL NF-κB inhibitor, CAPE, for 2 h and then treated with TNF-α for another 24 h, and miR-497 was detected by real-time qRT-PCR. (C) Two NF-κB binding sites were predicted in the promoter region of miR-497. Fragments containing all the wild-type binding sites (WT) or after deletion of the first binding site (WTΔ1) or the second binding site (WTΔ2) were cloned to pGL3 vector. (D) Luciferase activity was detected following transfection with different luciferase reporter vectors. (E) Gel-shift signals of two different NF-κB probes incubated with nuclear extracts of HepG2 cells. *$p < 0.05$; **$p < 0.01$; ***$p < 0.001$.

through direct targeting of SALL4.[27] Similarly, miR-219 serves a suppressive role in glioma growth and metastasis via targeting SALL4.[28] In a metastatic breast cancer model, SALL4 was also found to be a target of miR-33b.[29] In murine embryonic stem cells (ESCs), miR-294 and let-7c were reported to have opposing effects on SALL4 expression.[30] Another research in colorectal cancer show that miR-508 directly targets SALL4 and modulates its expression.[31] Recently, miR-98 was reported to regulate SALL4 in HCC.[32] However, other than this, little knowledge is available about the regulatory and interaction networks regarding the miRNAs and SALL4 in HCC. Our study shows that miR-497 regulates self-renewal and metastasis of HCC via targeting SALL4. More importantly, our study reveals a new signaling pathway that connected chronic inflammation with HCC stemness/EMT and explains how the micro-environment of chronic inflammation promoted HCC progression. Further investigation to identify new miRNAs that target SALL4 will further enrich our understanding on the regulation mechanisms of the HCC self-renewal.

Cancer stem cells are recognized as being responsible for metastasis and treatment resistance. The complicated cellular and molecular networks that regulate stemness properties and roles that inflammation might play in cancer progression are slowly being elucidated so far. Currently, STAT3 and NF-κB pathways are two major signaling pathways to link inflammation and cancer. Pro-inflammatory cytokines, such as IL-6 and TNF-α, which activate the transcription factors STAT3 and NF-κB, respectively, have been reported to be important for the development and progression of HCC.[33,34] Interestingly, Li et al. [35] have shown that chronic-inflammation-elicited liver progenitor cells could convert to liver cancer stem cells via the TNF-SRC-STAT3 signal, indicating the importance of chronic inflammation in the conversion of non-cancer cells to cancer stem cells. Kryczek et al.[36] have reported that T cell-derived IL-22 also promoted colorectal cancer stemness via STAT3-DOT1L axis. In the present study, we further confirmed that chronic inflammation participated in the stemness and metastatic potential maintenance in HCC cells via the miR-497-SALL4 signal for the first time.

In previous studies, there have been different reports on the regulation of miR-497 by the NF-κB signaling pathway. Wei et al.[37] observed that miR-195 and miR-497, which shared similar expression profiling, were negatively regulated by NF-κB in mice myoblast cells, while Mechtler et al.[38] reported that PIPK-1 and IL-1β, which activate NF-κB, can increase the miR-497 level. Our study further confirms that p65 can directly bind to a specific miR-497 promoter site and reduce its expression, which clarifies the negative regulation of miR-497 by NF-κB signaling.

In summary, our study identified the crucial roles of inflammatory micro-environment in stemness properties and metastatic potential maintenance; and more importantly, we elucidated a novel regulatory pathway of NF-κB/miR-497/SALL4 signaling that connected inflammation with self-renewal/metastasis phenotypes. These results could significantly expand our understanding of inflammation in tumor progression. Moreover, targeting the NF-κB/miR-497/SALL4 signaling axis might provide potential novel strategies for HCC patient treatment in the future.

MATERIALS AND METHODS

Cell Lines and Human Tissue Specimens

The HCC cell lines HepG2, Hep3B, and SK-Hep-1 and the HEK293T cell line were purchased from ATCC; the SMMC-7721, MHCC97-H, MHCC97-L, HCC-LM3, LO2, and Huh7 cell lines were purchased from the Cell Bank of the Shanghai Institute of Cell Biology (Shanghai, China). All cell lines were cultured in DMEM (Thermo Fisher Scientific) supplemented with 10% fetal bovine serum (FBS)

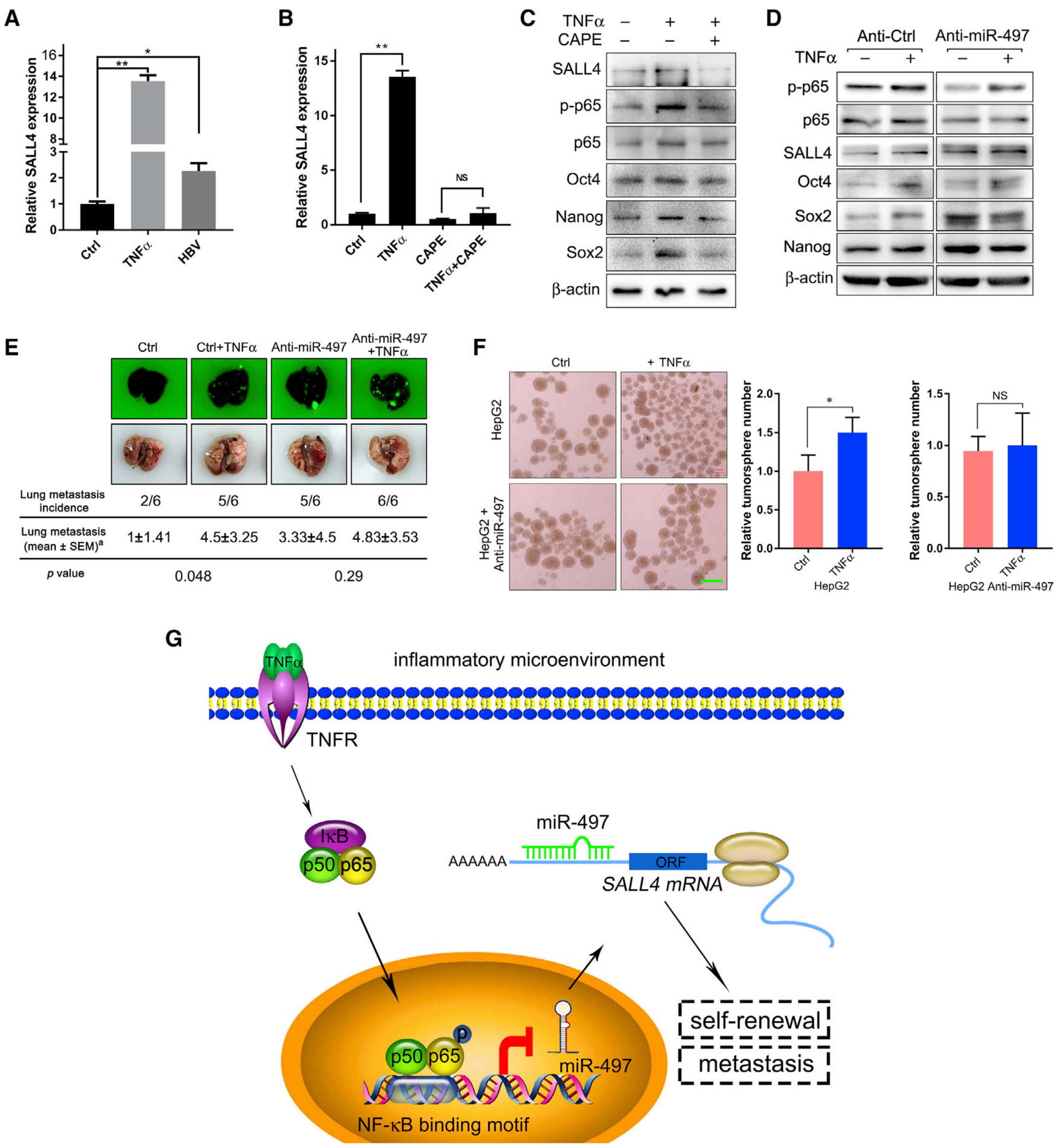

Figure 6. Inflammation Micro-environment Induces HCC Stemness through NF-κB/miR-497/SALL4 Axis

(A) Inflammation upregulated the mRNA level of SALL4. HepG2 cells were transfected with pAAV-HBV2.1 or treated with TNF-α, and the levels of SALL4 mRNA were analyzed using real-time qRT-PCR. (B) TNF-α upregulated SALL4 mRNA in an NF-κB-dependent manner. HepG2 cells were treated with CAPE and/or TNF-α as indicated, and the levels of SALL4 mRNA were analyzed using real-time qRT-PCR. (C) NF-κB inhibitor attenuated the induction function of TNF-α on SALL4 and stemness factor. HepG2 cells were treated with CAPE and/or TNF-α as indicated, and the expression of p-p65, p65, SALL4, Oct4, Sox2, and Nanog were detected by western blot. (D) miR-497 attenuated the induction function of TNF-α on SALL4 and stemness factor. Anti-Ctrl and Anti-miR-497 HepG2 cells were treated with TNF-α or not treated, and the expression of p-p65, p65, SALL4, Oct4, Sox2, and Nanog was detected by western blot. (E) miR-497 attenuated TNF-α treatment-induced incidence of lung metastasis in mice. NOD/SCID mice were injected with TNF-α pre-treated Anti-Ctrl or Anti-miR-497 SK-Hep-1 cells (1×10^6) via tail vein, followed by TNF-α treatment (0.5 mg/kg) for 3 weeks (twice per week). Representative images of lungs from one mouse per treatment group are shown. [a]Number of metastatic lesions were counted by detecting the fluorescence of GFP using the Molecular Imager ChemiDoc XRS System. (F) miR-497 attenuated TNF-α treatment-induced tumor sphere formation. Anti-Ctrl and Anti-miR-497 HepG2 cells were treated with TNF-α or not treated, and then the tumor-sphere-forming capacity was analyzed by tumor sphere formation assay. Scale bar, 400 μm. (G) A proposed model for inflammatory micro-environment contributes to self-renewal and metastasis features of HCC via the NF-κB/miR-497/SALL4 axis. *$p < 0.05$; **$p < 0.01$.

(Life Technologies), 100 IU penicillin, and 100 mg/mL streptomycin (Bio Basic).

Human HCC tissues were collected from 75 patients who underwent HCC resection at the Mengchao Hepatobiliary Hospital of Fujian Medical University from 2014 to 2015. The study was approved by the Medical Ethics Committee of Mengchao Hepatobiliary Hospital of Fujian Medical University. Written consent was received from all participants in this study at the time of surgery.

Plasmids and Reagents

miRNA mimics and inhibitors of hsa-miR-497 were purchased from RiboBio (RiboBio, Guangzhou, China). Stable knockdown transfectants of miR-497 were generated by lentiviral transduction using pMIRZip plasmid (System Biosciences, Palo Alto, CA, USA). Lentiviral vectors expressing scrambled shRNA or SALL4-specific shRNA were purchased from OriGene.

SALL4 3′ UTR luciferase reporter plasmid was constructed for miR-497 target analysis. The 3′ UTR segment of SALL4, including the wild-type sequence and mutant sequence, were synthesized by GENEWIZ (Suzhou, China) and inserted into the pMIR-REPORT vector. The sequences of the insertion fragment are listed in Table S1.

The fragments of the promoter region of miR-497 containing the predicted p65-binding site were synthesized by GENEWIZ and inserted into the PGL3 vector (Promega). The sequences of the insertion fragment were listed in Table S1.

Luciferase Reporter Assay

Luciferase reporter assay was performed as described previously.[39] Briefly, 293T cells were transfected with luciferase reporter constructs, β-gal, and other different genes as required using Lipofectamine 3000 (Invitrogen) in a 24-well plate. The cells were harvested after transfection for 24 or 48 h, and the luciferase activity was measured using the Bright-Glo Luciferase Assay System (Promega, Fitchburg, WI, USA) according to the manufacturer's instructions. β-galactosidase (β-gal) activities were measured as the internal control. Data were obtained by normalization of β-gal activity to luciferase activity. The bars represent the mean ± SEM from three independent experiments.

Western Blot Analysis

Cells were harvested and lysed in RIPA supplemented with protease inhibitors. Equal amounts of protein lysates were separated by SDS-PAGE and electrically transferred to nitrocellulose membrane. The membranes were probed with the following specific primary antibodies: SALL4 (Abnova, H00057167-M03), E-cadherin (Cell Signaling Technology, #3195), vimentin (Cell Signaling Technology, #5741), Oct4 (Abcam, ab18976), Bmi-1 (Abcam, ab126783), Nanog (Cell Signaling Technology, #4903), NF-κB p65 (Cell Signaling Technology, #8242), Phospho-NF-κB p65 (Cell Signaling Technology, #3033), Sox2 (Cell Signaling Technology, #14962), and β-actin (Cell Signaling Technology, #12262). Then they were blotted with an HRP (horseradish peroxidase)-conjugated secondary antibody. Blots were visualized by enhanced chemiluminescence (ECL).

Lentiviral Transduction to Stably Down-Regulate miR-497 Expression in HCC Cells

The lentivector-based anti-miRNA expression vector, pmiRZip, was purchased from System Biosciences (Mountain View, CA, USA). To generate the miR-497-ZIP anti-miR-497 lentiviral construct, the specific anti-miR-497 sequence was subcloned into pmiRZip (sense: 5′-GATCCCTCGTCGTCACTGTGGTTTGTCTTCCTGTCAGAACAAACCACAGTGTGCTGCTGTTTTTG-3′; anti-sense: 5′- AATTCAAAAACAGCAGCACACTGTGGTTTGTTCTGACAGGAAGACAAACCACAGTGTCGACGAGG-3′). The miR-497-ZIP or miRZip control vector was transduced into HCC cells according to the manufacturer's protocol.

Real-Time RT-PCR

Total RNA extraction from cell lines or frozen tissues was performed by using TRIzol reagent (Invitrogen, Carlsbad, CA, USA), and cDNA was synthesized using the Transcriptor First Strand cDNA Synthesis Kit (Roche, Mannheim, Germany) for mRNA analysis. The miRcute miRNA First-strand cDNA Synthesis Kit (Tiangen, Beijing, China) was used for miRNA analysis. Real-time PCR assay for mRNA analysis was performed by using the SYBR Green ER qPCR Super Mix Universal Kit (Invitrogen), and miRNA analysis was performed by using the miRcute miRNA qPCR Detection Kit (Tiangen, Beijing, China) in the StepOnePlus Real-Time PCR System (Applied Biosystems). Primers used for real-time PCR are listed in Table S2. The primer for miR-497 was purchased from Tiangen (Beijing, China). U6 and GAPDH were used as controls for the detection of miRNA and mRNA, respectively.

Tumor Spheroid Formation Assay

Cells were plated in ultralow-attachment six-well plates (Corning, Corning, NY, USA) at a density of 1×10^4 cells per milliliter in serum-free DMEM/F12 medium (Invitrogen, Carlsbad, CA, USA) supplemented with 20 ng/mL epidermal growth factor (Sigma, USA), 10 ng/mL basic fibroblast growth factor (Sigma), 5 mg/mL insulin (Sigma), 1 × B27 supplement (Invitrogen), and 0.4% BSA (Sigma). Cells were cultured under 5% CO_2 at 37°C for a week.

Transwell Invasion, Migration, and Wound Healing Assays

Cell migration was assayed using the Transwell method, with 8-μm pore filters (Corning, Corning, NY, USA). The lower chamber was filled with DMEM, supplemented with 10% FBS, and 2×10^4 cells in 0.5 mL DMEM were loaded into the upper chamber. After a 22-h incubation period, the cells that migrated to the bottom of the membrane were fixed with 4% formaldehyde. The cells on the top of the membrane were removed by wiping the surface with a cotton swab. The cells were stained with 0.5% crystal violet and observed under a microscope. The number of migrated cells was counted at a magnification of 200× from five adjacent microscope fields. For the Matrigel invasion assay, the procedures used were the same as those described earlier, except that the Transwell membrane was coated

with Matrigel (BD Biosciences, San Jose, CA, USA) to form a matrix barrier.

Wound healing assay was carried out using culture inserts (IBIDI, Gräfelfing, Germany). Cells were seeded into culture inserts in a 6-well plate and were incubated to allow them to adhere. The culture insert provided two cell-culture reservoirs that were separated by approximately a 0.5-mm-thick wall. The culture insert was removed after the cells were cultured to full confluence, and a "wound" of approximately 0.5 mm was formed between the two cell patches. Wound closure was observed after 24 h and was photographed under a microscope. The fraction of cell coverage across the line represents the migration rate.

Xenograft Assay

The animal experiments were approved by Fuzhou Medical Experimental Animal Care Commission. All mice were housed and maintained under pathogen-free conditions in accordance with the institutional guidelines of the Experimental Animal Center of Fuzhou General Hospital. Six- to eight-week-old female non-obese diabetic/severe combined immunodeficiency (NOD/SCID) mice were used for this experiment. First, 1×10^6 SK-Hep-1 and SMMC-7721 cells stably transduced with miRZip-497 (anti-miR-497) or miRZip control vector were injected through the tail vein of each mouse. Then, the injected mice were sacrificed at week 6 post-inoculation, and the tumor incidence was assessed by detecting the fluorescence of GFP using the Molecular Imager ChemiDoc XRS System (Bio-Rad, Hercules, CA, USA).

For TNF-α treatment assay, the SK-Hep-1 cells (anti-miR497 or miRZip control) were pre-treated with 200 ng/mL TNF-α for 48 h and then injected through the tail vein of each mouse (1×10^6 cells); then the mice were treated twice per week with intraperitoneal injections of PBS or TNF-α (0.5 mg/kg) for 3 weeks. Afterward, the mice were sacrificed, and tumor incidences were detected at week 4.

Histology and Staining

Lung and liver tissues were collected and fixed for 24 h in formalin and then paraffin embedded and sectioned into slices. Tissue sections were stained with H&E or were immune-stained using the following corresponding antibodies: Sall4 (Abnova, H00057167-M03, 1:800), Nanog (Cell Signaling Technology, #4903, 1:800), N-cadherin (Cell Signaling Technology, #13116, 1:200), E-cadherin (Cell Signaling Technology, #3195, 1:200), and OCT4 (Abcam, ab18976, 1:100).

EMSA

EMSA was performed by using the LightShift Chemiluminescent EMSA Kit (Thermo Fisher Scientific) according to the manual's description. Nuclear extracts were isolated as described previously.[40] The probes were synthesized and double labeled with biotin. The sequences of the probes were as follows: probe 1, 5′-ACCCCACCCTAG GGATCCCCTGAGCTGAGTT-3′ and 5′- AACTCAGCTCAGGGGA TCCCTAGGGTGGGGT-3′; probe 2, 5′- AGGTGGTGCTGGGGTCT TCCCAGCACTGC-3′ and 5′-GCAGTGCTGGGAAGACCCCAGCA CCACCT-3′.

Statistical Analysis

Statistical analysis was performed using SPSS v19.0. Statistical analyses of normally distributed variables were performed using the Student's t test, and analyses of data with skewed distributions were performed using the Mann-Whitney U test. The results are expressed as the mean ± SEM. Pearson's correlation analysis was conducted to assess the correlation statistics between two variables. For the clinical-pathological analysis, the chi-square test was performed. Survival curves were constructed using the Kaplan-Meier method and evaluated using the log-rank test. The Cox proportional hazard regression model was used to identify factors that were independently associated with OS. $p < 0.05$ was considered as statistically significant.

SUPPLEMENTAL INFORMATION

Supplemental Information can be found online at https://doi.org/10.1016/j.omto.2019.08.009.

AUTHOR CONTRIBUTIONS

B.Z., A.H., and X.L. designed research. B.Z., Y.W., X.T., K.K., X.Z., F.W., S.L., and Y.S. performed the experiments with data analysis; N.L., Z.C., Y.Z., Y.L., L.W., Q.L., and J.L. provided support with experimental materials and techniques. B.Z. and X.L. wrote the paper.

CONFLICTS OF INTEREST

The authors declare no competing interests.

ACKNOWLEDGMENTS

This work was supported by the National Natural Science Foundation of China (nos. 81672376 and 81602102); the Medical Innovation Project of Fujian Province (2016-CX-48); the Scientific Foundation of Fuzhou City (2015-S-143-19); the Natural Science Foundation of Fujian Province (2016J01417); the Fuzhou Health and Family Planning Science and Technology Project (2017-S-wt2); the Young and Middle-Aged Talent Training Project of Fujian Provincial Health and Family Planning Commission (nos. 2018-ZQN-76, 2018-ZQN-37, and 2016-1-44); and the Joint Funds for the Innovation of Science and Technology of Fujian Province (nos. 2017Y9116 and 2017Y9041).

REFERENCES

1. Torre, L.A., Bray, F., Siegel, R.L., Ferlay, J., Lortet-Tieulent, J., and Jemal, A. (2015). Global cancer statistics, 2012. CA Cancer J. Clin. *65*, 87–108.
2. Clarke, M.F., and Fuller, M. (2006). Stem cells and cancer: two faces of eve. Cell *124*, 1111–1115.
3. Kim, H., and Park, Y.N. (2014). Hepatocellular carcinomas expressing 'stemness'-related markers: clinicopathological characteristics. Dig. Dis. *32*, 778–785.
4. Grivennikov, S.I., Greten, F.R., and Karin, M. (2010). Immunity, inflammation, and cancer. Cell *140*, 883–899.
5. Zhang, J., Tam, W.L., Tong, G.Q., Wu, Q., Chan, H.Y., Soh, B.S., Lou, Y., Yang, J., Ma, Y., Chai, L., et al. (2006). Sall4 modulates embryonic stem cell pluripotency and early embryonic development by the transcriptional regulation of Pou5f1. Nat. Cell Biol. *8*, 1114–1123.

6. Yang, J., Chai, L., Fowles, T.C., Alipio, Z., Xu, D., Fink, L.M., Ward, D.C., and Ma, Y. (2008). Genome-wide analysis reveals Sall4 to be a major regulator of pluripotency in murine-embryonic stem cells. Proc. Natl. Acad. Sci. USA *105*, 19756–19761.

7. Oikawa, T., Kamiya, A., Zeniya, M., Chikada, H., Hyuck, A.D., Yamazaki, Y., Wauthier, E., Tajiri, H., Miller, L.D., Wang, X.W., et al. (2013). Sal-like protein 4 (SALL4), a stem cell biomarker in liver cancers. Hepatology *57*, 1469–1483.

8. Yong, K.J., Gao, C., Lim, J.S., Yan, B., Yang, H., Dimitrov, T., Kawasaki, A., Ong, C.W., Wong, K.F., Lee, S., et al. (2013). Oncofetal gene SALL4 in aggressive hepatocellular carcinoma. N. Engl. J. Med. *368*, 2266–2276.

9. Zeng, S.S., Yamashita, T., Kondo, M., Nio, K., Hayashi, T., Hara, Y., Nomura, Y., Yoshida, M., Hayashi, T., Oishi, N., et al. (2014). The transcription factor SALL4 regulates stemness of EpCAM-positive hepatocellular carcinoma. J. Hepatol. *60*, 127–134.

10. Shibahara, J., Ando, S., Hayashi, A., Sakamoto, Y., Hesegawa, K., Kokudo, N., and Fukayama, M. (2014). Clinicopathologic characteristics of SALL4-immunopositive hepatocellular carcinoma. Springerplus *3*, 721.

11. Bartel, D.P. (2004). MicroRNAs: genomics, biogenesis, mechanism, and function. Cell *116*, 281–297.

12. Garofalo, M., and Croce, C.M. (2015). Role of microRNAs in maintaining cancer stem cells. Adv. Drug Deliv. Rev. *81*, 53–61.

13. Calin, G.A., Ferracin, M., Cimmino, A., Di Leva, G., Shimizu, M., Wojcik, S.E., Iorio, M.V., Visone, R., Sever, N.I., Fabbri, M., et al. (2005). A microRNA signature associated with prognosis and progression in chronic lymphocytic leukemia. N. Engl. J. Med. *353*, 1793–1801.

14. He, L., Thomson, J.M., Hemann, M.T., Hernando-Monge, E., Mu, D., Goodson, S., Powers, S., Cordon-Cardo, C., Lowe, S.W., Hannon, G.J., and Hammond, S.M. (2005). A microRNA polycistron as a potential human oncogene. Nature *435*, 828–833.

15. Ma, L., Teruya-Feldstein, J., and Weinberg, R.A. (2007). Tumour invasion and metastasis initiated by microRNA-10b in breast cancer. Nature *449*, 682–688.

16. Yakaboski, E., Jares, A., and Ma, Y. (2014). Stem cell gene SALL4 in aggressive hepatocellular carcinoma: a cancer stem cell-specific target? Hepatology *60*, 419–421.

17. Yan, J.J., Zhang, Y.N., Liao, J.Z., Ke, K.P., Chang, Y., Li, P.Y., Wang, M., Lin, J.S., and He, X.X. (2015). MiR-497 suppresses angiogenesis and metastasis of hepatocellular carcinoma by inhibiting VEGFA and AEG-1. Oncotarget *6*, 29527–29542.

18. Ding, W.Z., Ni, Q.F., Lu, Y.T., Kong, L.L., Yu, J.J., Tan, L.W., and Kong, L.B. (2016). MicroRNA-497 regulates cell proliferation in hepatocellular carcinoma. Oncol. Lett. *11*, 1081–1088.

19. Ringelhan, M., Pfister, D., O'Connor, T., Pikarsky, E., and Heikenwalder, M. (2018). The immunology of hepatocellular carcinoma. Nat. Immunol. *19*, 222–232.

20. Seki, E., and Schwabe, R.F. (2015). Hepatic inflammation and fibrosis: functional links and key pathways. Hepatology *61*, 1066–1079.

21. Budhu, A., Jia, H.L., Forgues, M., Liu, C.G., Goldstein, D., Lam, A., Zanetti, K.A., Ye, Q.H., Qin, L.X., Croce, C.M., et al. (2008). Identification of metastasis-related microRNAs in hepatocellular carcinoma. Hepatology *47*, 897–907.

22. Ladeiro, Y., Couchy, G., Balabaud, C., Bioulac-Sage, P., Pelletier, L., Rebouissou, S., and Zucman-Rossi, J. (2008). MicroRNA profiling in hepatocellular tumors is associated with clinical features and oncogene/tumor suppressor gene mutations. Hepatology *47*, 1955–1963.

23. Toffanin, S., Hoshida, Y., Lachenmayer, A., Villanueva, A., Cabellos, L., Minguez, B., Savic, R., Ward, S.C., Thung, S., Chiang, D.Y., et al. (2011). MicroRNA-based classification of hepatocellular carcinoma and oncogenic role of miR-517a. Gastroenterology *140*, 1618–1628.e16.

24. Guo, S.T., Jiang, C.C., Wang, G.P., Li, Y.P., Wang, C.Y., Guo, X.Y., Yang, R.H., Feng, Y., Wang, F.H., Tseng, H.Y., et al. (2013). MicroRNA-497 targets insulin-like growth factor 1 receptor and has a tumour suppressive role in human colorectal cancer. Oncogene *32*, 1910–1920.

25. Xie, Y., Wei, R.R., Huang, G.L., Zhang, M.Y., Yuan, Y.F., and Wang, H.Y. (2014). Checkpoint kinase 1 is negatively regulated by miR-497 in hepatocellular carcinoma. Med. Oncol. *31*, 844.

26. Li, D., Zhao, Y., Liu, C., Chen, X., Qi, Y., Jiang, Y., Zou, C., Zhang, X., Liu, S., Wang, X., et al. (2011). Analysis of miR-195 and miR-497 expression, regulation and role in breast cancer. Clin. Cancer Res. *17*, 1722–1730.

27. He, J., Zhang, W., Zhou, Q., Zhao, T., Song, Y., Chai, L., and Li, Y. (2013). Low-expression of microRNA-107 inhibits cell apoptosis in glioma by upregulation of SALL4. Int. J. Biochem. Cell Biol. *45*, 1962–1973.

28. Jiang, B., Li, M., Ji, F., and Nie, Y. (2017). MicroRNA-219 exerts a tumor suppressive role in glioma via targeting Sal-like protein 4. Exp. Ther. Med. *14*, 6213–6221.

29. Lin, Y., Liu, A.Y., Fan, C., Zheng, H., Li, Y., Zhang, C., Wu, S., Yu, D., Huang, Z., Liu, F., et al. (2015). MicroRNA-33b inhibits breast cancer metastasis by targeting HMGA2, SALL4 and Twist1. Sci. Rep. *5*, 9995.

30. Melton, C., Judson, R.L., and Blelloch, R. (2010). Opposing microRNA families regulate self-renewal in mouse embryonic stem cells. Nature *463*, 621–626.

31. Yan, T.T., Ren, L.L., Shen, C.Q., Wang, Z.H., Yu, Y.N., Liang, Q., Tang, J.Y., Chen, Y.X., Sun, D.F., Zgodzinski, W., et al. (2018). miR-508 defines the stem-like/mesenchymal subtype in colorectal cancer. Cancer Res. *78*, 1751–1765.

32. Zhou, W., Zou, B., Liu, L., Cui, K., Gao, J., Yuan, S., and Cong, N. (2016). MicroRNA-98 acts as a tumor suppressor in hepatocellular carcinoma via targeting SALL4. Oncotarget *7*, 74059–74073.

33. Naugler, W.E., Sakurai, T., Kim, S., Maeda, S., Kim, K., Elsharkawy, A.M., and Karin, M. (2007). Gender disparity in liver cancer due to sex differences in MyD88-dependent IL-6 production. Science *317*, 121–124.

34. Subramaniam, A., Shanmugam, M.K., Perumal, E., Li, F., Nachiyappan, A., Dai, X., Swamy, S.N., Ahn, K.S., Kumar, A.P., Tan, B.K., et al. (2013). Potential role of signal transducer and activator of transcription (STAT)3 signaling pathway in inflammation, survival, proliferation and invasion of hepatocellular carcinoma. Biochim. Biophys. Acta *1835*, 46–60.

35. Li, X.F., Chen, C., Xiang, D.M., Qu, L., Sun, W., Lu, X.Y., Zhou, T.F., Chen, S.Z., Ning, B.F., Cheng, Z., et al. (2017). Chronic inflammation-elicited liver progenitor cell conversion to liver cancer stem cell with clinical significance. Hepatology *66*, 1934–1951.

36. Kryczek, I., Lin, Y., Nagarsheth, N., Peng, D., Zhao, L., Zhao, E., Vatan, L., Szeliga, W., Dou, Y., Owens, S., et al. (2014). IL-22(+)CD4(+) T cells promote colorectal cancer stemness via STAT3 transcription factor activation and induction of the methyltransferase DOT1L. Immunity *40*, 772–784.

37. Wei, W., Zhang, W.Y., Bai, J.B., Zhang, H.X., Zhao, Y.Y., Li, X.Y., and Zhao, S.H. (2016). The NF-κB-modulated microRNAs miR-195 and miR-497 inhibit myoblast proliferation by targeting Igf1r, Insr and cyclin genes. J. Cell Sci. *129*, 39–50.

38. Mechtler, P., Singhal, R., Kichina, J.V., Bard, J.E., Buck, M.J., and Kandel, E.S. (2015). MicroRNA analysis suggests an additional level of feedback regulation in the NF-κB signaling cascade. Oncotarget *6*, 17097–17106.

39. Zhao, B., Zhao, W., Wang, Y., Xu, Y., Xu, J., Tang, K., Zhang, S., Yin, Z., Wu, Q., and Wang, X. (2015). Connexin32 regulates hepatoma cell metastasis and proliferation via the p53 and Akt pathways. Oncotarget *6*, 10116–10133.

40. Zhao, B.X., Chen, H.Z., Du, X.D., Luo, J., He, J.P., Wang, R.H., Wang, Y., Wu, R., Hou, R.R., Hong, M., and Wu, Q. (2011). Orphan receptor TR3 enhances p53 transactivation and represses DNA double-strand break repair in hepatoma cells under ionizing radiation. Mol. Endocrinol. *25*, 1337–1350.

OMTO, Volume *15*

Supplemental Information

Inflammatory Micro-environment Contributes to Stemness Properties and Metastatic Potential of HCC via the NF-κB/miR-497/SALL4 Axis

Bixing Zhao, Yingchao Wang, Xionghong Tan, Kun Ke, Xiaoyuan Zheng, Fei Wang, Shubing Lan, Naishun Liao, Zhixiong Cai, Yingjun Shi, Youshi Zheng, Yongping Lai, Lili Wang, Qin Li, Jingfeng Liu, Aimin Huang, and Xiaolong Liu

Supplementary information

Supplementary Figures

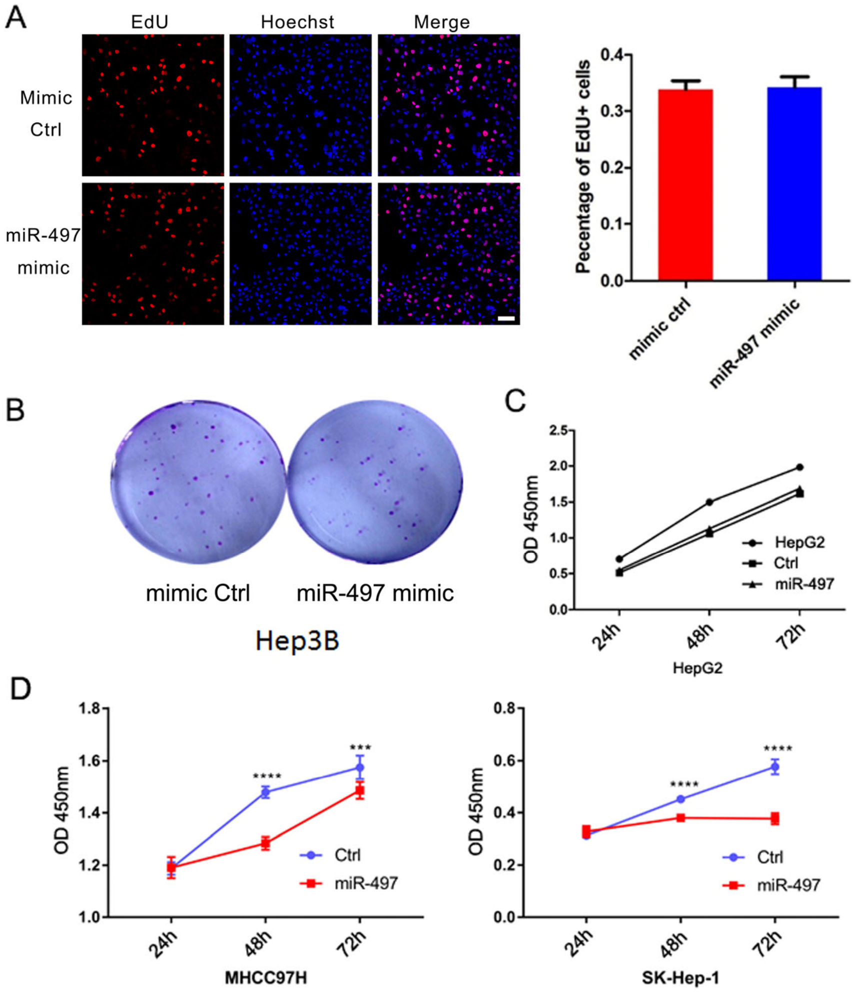

Figure S1. Effect of miR-497 on proliferation of hepatocellular carcinoma cells. (A) Hep3B cells were transfected with mimic Control or miR-497 mimic. The EdU proliferation assay was performed 48 h after transfection. Representative image and ratio of EdU-positive Hep3B cells were shown. Scale bar, 100μm. (B) The colony formation assay was conducted to determine the colony-forming growth of cells and the colonies were captured on the 14th day after seeding. (C-D) Proliferation was analyzed by CCK-8 assay in miR-497 over-expressing HepG2 (C) MHCC97H (D) and SK-Hep-1 (D) cells. ***P<0.001, ****P<0.0001.

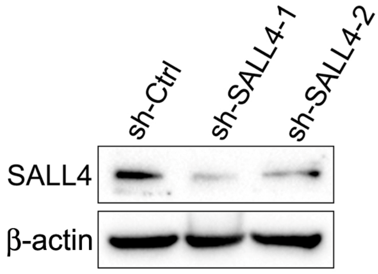

Figure S2. SALL4 knockdown verification at the protein level by western blot analysis. Hep3B cells were infected with lentiviruses to introduce constitutively active shRNA control or shRNA against SALL4, and western blot was carried out 4 days after infection.

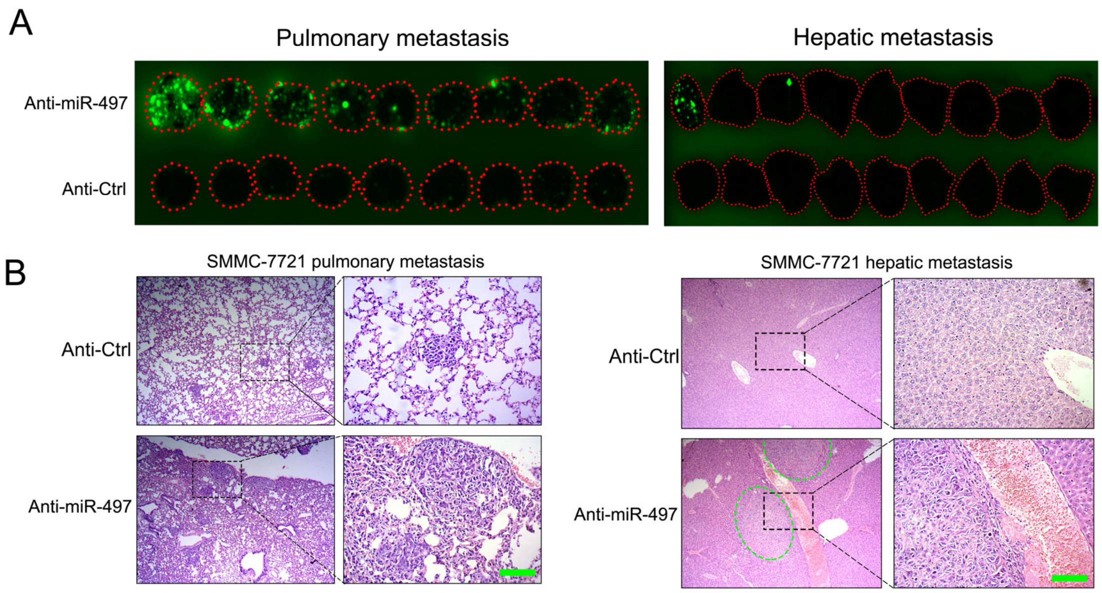

Figure S3. miR-497 suppressed SMMC-7721 cell lung and hepatic metastasis in mouse model (A) Lung and hepatic metastasis of anti-control and anti-miR497 SMMC-7721 cells in NOD/SCID mice at 6 weeks after tail vein implantation. Lungs and livers from the NOD/SCID mice were dissected and the presence of HCC metastasis and detected by green fluorescence signal. (B) Representative H&E-stained sections of the lung and liver tissues collected from anti-Ctrl and Anti-miR-497 groups. Scale bars, 100μm.

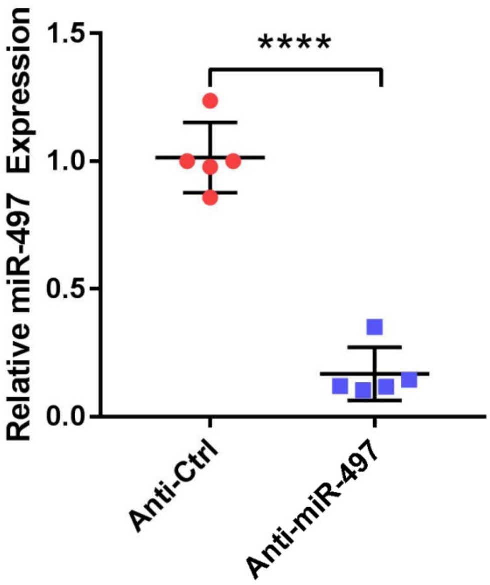

Figure S4. miR-497 expression verification in lung metastasis nodules. Lung metastasis nodules from the NOD/SCID mice (Anti-Ctrl and Anti-miR-497 group) were dissected and the levels of miR-497 were analyzed using real-time qRT-PCR. ****$P<0.0001$.

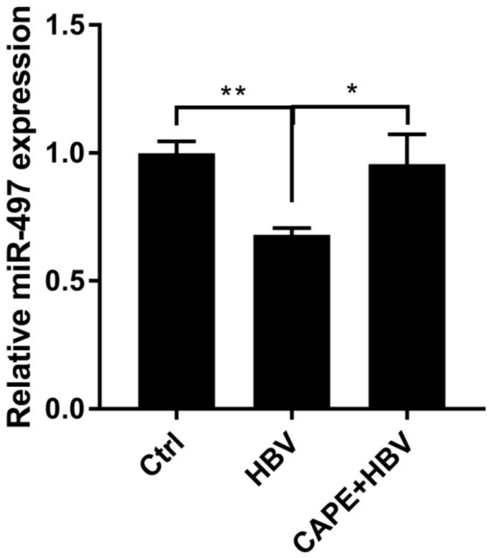

Figure S5. HBV suppresses miR-497 expression in a NF-κB dependent manner. HepG2 cells were transfected with pAAV-HBV2.1, then treated with 25μg/ml of CAPE, for 24 h, and miR-497 was detected by real-time qRT-PCR. *P<0.05, **P<0.01.

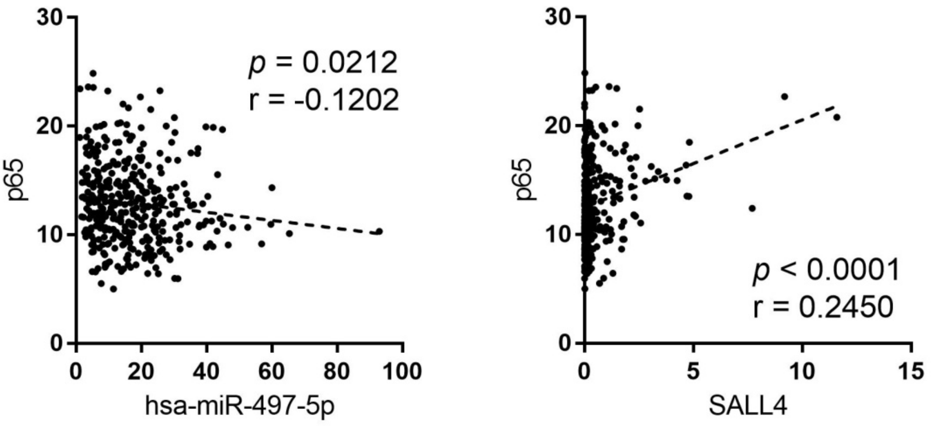

Figure S6. TCGA data analysis of the correlation of p65 with miR-497 and p65 with SALL4. Inverse correlation of miR-497 expression with p65 expression. Positive correlation of SALL4 expression with p65 expression.

Supplementary Table 1. Sequences of the insertion fragments of Luciferase reporter plasmid.

Name	WT/Mutant	Insertion sequence
SALL4 3’-UTR luciferase reporter	WT	GGGAGAACTTGCGTGGAAGGAGCAATGCAGACACAGTGAAATCTCTAGAATCTGCTTTGTTTTGTAAGAACTCATCTCCTCCTGTTTTCTTTTTCTTACTGATATGCAAATGATGTTTACTACGTTGGTTGTGACCACAACCTCAGGCAAGTGCTACAATCACGATTGTTGCTATGCTGCTTTGCAAAAAGTTGAAAAAATAAAAAAAAAATGCATACCA AAAA
	Mutant	GGGAGAACTTGCGTGGAAGGAGCAATGCAGACACAGTGAAATCTCTAGAATCTGCTTTGTTTTGTAAGAACTCATCTCCTCCTGTTTTCTTTTTCTTACTGATATGCAAATGATGTTTACTACGTTGGTTGTGACCACAACCTCAGGCAAGTGCTACAATCACGATTGTTGCTAACGACGATTGCAAAAAGTTGAAAAAATAAAAAAAAAATGCATACCAAAAA
PGL3-miR497-promoter	WT	CCCCACCCTAGGGATCCCCTGAGCTGAGTTCCTACAGAGGGAAGATGGTCCAATCTTACTACACTGTGAGCTCATCCCCATGGTCCGTCGCCTTCCAGTTGCCTGCTCAGCCCGTCCCTGGTTCCTCCCAAACGTTTTTGGGGGCCATGTTTGCCTTTTAAGGCTTCTCTATCCCCCCGCTCCTGGAGGTGGTGCTGGGGTCTTCCCAGCACTGC
	D1	CCCCACCCTATGAGCTGAGTTCCTACAGAGGGAAGATGGTCCAATCTTACTACACTGTGAGCTCATCCCCATGGTCCGTCGCCTTCCAGTTGCCTGCTCAGCCCGTCCCTGGTTCCTCCCAAACGTTTTTGGGGGCCATGTTTGCCTTTTAAGGCTTCTCTATCCCCCCGCTCCTGGAGGTGGTGCTGGGGTCTTCCCAGCACTGC
	D2	CCCCACCCTAGGGATCCCCTGAGCTGAGTTCCTACAGAGGGAAGATGGTCCAATCTTACTACACTGTGAGCTCATCCCCATGGTCCGTCGCCTTCCAGTTGCCTGCTCAGCCCGTCCCTGGTTCCTCCCAAACGTTTTTGGGGGCCATGTTTGCCTTTTAAGGCTTCTCTATCCCCCCGCTCCTGGAGGTGGTGCTCCAGCACTGC

Supplementary Table 2. Primers for qRT-PCR

Name	F/R	Primer Sequence
SALL4	F	CGCCCCGTGTGTCATGTAGTGAAC
	R	TCCGAGAACAGCCGCACTGAGATGGAAG
CDH1	F	GCCGAGAGCTACACGTTCA
	R	GACCGGTGCAATCTTCAAA
Vimentin	F	GACGCCATCAACACCGAGTT
	R	CTTTGTCGTTGGTTAGCTGGT
Nanog	F	CTATAACTGTGGAGAGGAAT
	R	AGTGGTCTGCTGTATTAC
Oct4	F	GTATTCAGCCAAACGACCAT
	R	CTTCCTCCACCCACTTCT
Bmi-1	F	GCTTTGTGGAGGGTACTTCATT
	R	AGGACAATACTTGCTGGTCTC
GAPDH	F	AGCCACATCGCTCAGACAC
	R	GCCCAATACGACCAAATCC

Supplementary Table 3: Univariate and Multivariate Analysis of Factors Associated with Overall survival.

**Clinical Variables**	**Case Number**	**Univariate analysis**		**Multivariate analysis**	
		**HR (95% CI)**	***P* value**	**HR (95% CI)**	***P* value**
miR-497 (Low vs High)	38/37	0.388 (0.160-0.945)	**0.037**	0.286 (0.112-0.730)	**0.009**
Gender (M vs F)	68/7	2.621 (0.353-19.452)	0.346		
Age-yr (≥ 55 vs < 55)	36/39	1.460 (0.640-3.331)	0.369		
Tumor size (> 5 vs ≤ 5 cm)	26/49	5.519 (2.328-13.087)	**0.001**	5.040 (2.075-12.240)	**0.001**
Vascular invasion (Yes vs No)	44/31	0.522 (0.214-1.269)	0.151		
AFP (> 400 vs ≤ 400 ng/ml)	21/54	3.145 (1.384-7.147)	**0.006**	2.618 (1.091-6.284)	**0.031**
HBV (Positive vs Negative)	68/7	0.405 (0.055-3.003)	0.376		
Tumor number (Solitary vs Multiple)	60/15	1.372 (0.541-3.480)	0.506		
Cirrhosis (Yes vs No)	69/6	2.514 (0.745-8.486)	0.138		
TNM stage (I/II vs III/IV)	55/20	3.578 (1.574-8.130)	**0.002**	1.743 (0.682-4.451)	0.246
